# Second-Shell Basic Residues Expand the Two-Metal-Ion Architecture of DNA and RNA Processing Enzymes

**DOI:** 10.1016/j.str.2017.11.008

**Published:** 2018-01-02

**Authors:** Vito Genna, Matteo Colombo, Marco De Vivo, Marco Marcia

**Affiliations:** 1 Laboratory of Molecular Modelling & Drug Discovery, Istituto Italiano di Tecnologia, Via Morego 30, 16163, Genoa, Italy; 2 European Molecular Biology Laboratory, Grenoble Outstation, 71 Avenue des Martyrs, Grenoble 38042, France; 3 IAS-5/INM-9 Computational Biomedicine Forschungszentrum Jülich, Wilhelm-Johnen-Straße, 52428 Jülich, Germany

**Keywords:** ribozymes, splicing, gene editing, DNA/RNA polymerases, enzyme engineering, molecular simulations, molecular dynamics, metalloenzyme

## Abstract

Synthesis and scission of phosphodiester bonds in DNA and RNA regulate vital processes within the cell. Enzymes that catalyze these reactions operate mostly via the recognized two-metal-ion mechanism. Our analysis reveals that basic amino acids and monovalent cations occupy structurally conserved positions nearby the active site of many two-metal-ion enzymes for which high-resolution (<3 Å) structures are known, including DNA and RNA polymerases, nucleases such as Cas9, and splicing ribozymes. Integrating multiple-sequence and structural alignments with molecular dynamics simulations, electrostatic potential maps, and mutational data, we found that these elements always interact with the substrates, suggesting that they may play an active role for catalysis, in addition to their electrostatic contribution. We discuss possible mechanistic implications of this expanded two-metal-ion architecture, including inferences on medium-resolution cryoelectron microscopy structures. Ultimately, our analysis may inspire future experiments and strategies for enzyme engineering or drug design to modulate nucleic acid processing.

## Highlights

• Basic residues in the active site of two-metal-ion enzymes are structurally conserved
• These residues are also conserved in evolution
• Mutagenesis suggests these residues may exert an effect on DNA- and RNA processing
• Our work offers insights into CRISPR/Cas9, spliceosome, and DNA/RNA polymerases

## Graphical Abstract

**Figure:**
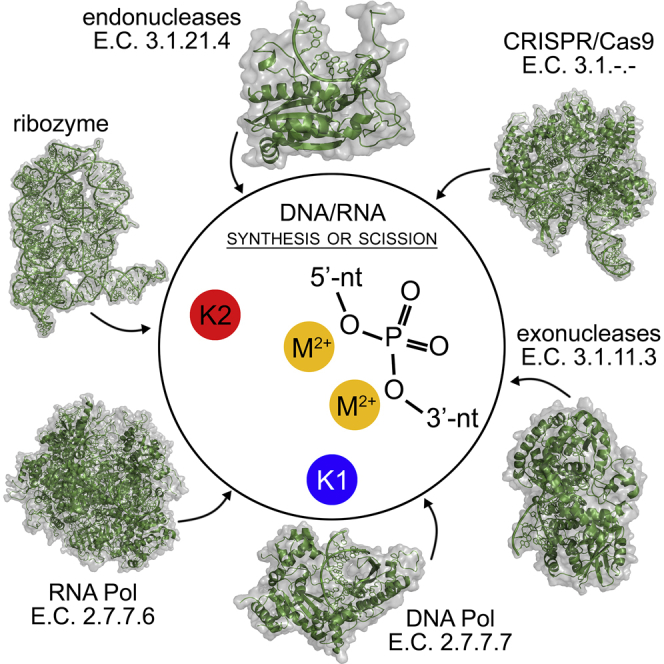

## Teaser

Genna et al. reveal structural conservation of basic amino acids and monovalent cations in the active site of two-metal-ion enzymes and ribozymes, which catalyze the vital biological reactions of DNA and RNA synthesis and scission. The work offers insights into fundamental two-metal-ion machineries such as CRISPR/Cas9, the spliceosome, and DNA/RNA polymerases.

## Introduction

Enzymatic cleavage and formation of phosphodiester bonds in DNA and RNA is central to life and health. These reactions allow processing nucleic acids during DNA replication, DNA recombination, DNA repair, transcription, splicing, and defense from pathogens (Yang et al., 2006). All these key chemical processes are controlled by vital cellular machineries including both protein and RNA enzymes (Strater et al., 1996), such as endo- and exonucleases, DNA and RNA polymerases, and ribozymes (i.e., group II intron and spliceosome) (Table 1). Independent of their biopolymer scaffold, these cellular machineries display a surprising degree of structural similarity (Livesay et al., 2003, Yang et al., 2006), suggesting convergence of their enzymatic reaction mechanism. Indeed, Steitz and Steitz (1993) first described the so-called two-metal-ion mechanism, which is shared among most nucleic acid-processing enzymes. According to this general mechanism, divalent metal ions (typically Mg, Mn, and Zn) are chelated by acidic groups of strictly conserved catalytic residues and the phosphodiester substrate is properly oriented via three specific binding sites (sites 1, 2, and 3) (Steitz and Steitz, 1993). Then, one metal (usually referred to as metal A, M_A_) stabilizes the activated nucleophile, while the second metal (metal B, M_B_) assists the release of the leaving group. Together, M_A_ and M_B_ stabilize the trigonal bipyramidal intermediate formed at the transition state (Steitz and Steitz, 1993). Notably, some enzymes such as the ββα-Me and HNH nucleases may operate with only one catalytic metal ion (M_A_, which is structurally conserved), while a basic amino acid replaces the missing M_B_ for catalysis (Yang, 2008). In all cases, a first-shell structural architecture centered on two conserved and positively charged elements located in the catalytic site is crucial for efficient DNA and RNA processing (Palermo et al., 2015).

**Table 1:** Representative Two-Metal-Ion Enzymes that Possess Positively Charged Elements in the Second Coordination Shell of the Active Site

Enzyme	PDB ID	Resolution (Å)	Enzymatic Classification (E.C.)	K1	d1 (Å)^a^	d-ac (Å)^b^	Mutants	Functional Defect	K2	d2 (Å)^a^	d-Sub (Å)^c^	Mutants	Functional Defect
Group II intron (*O. iheyensis*)	4FAR	2.86	ribozyme	K1	3.83	2.60	K to Li, Na, Cs (Marcia and Pyle, 2012)	distortion of active site, splicing defect	K2	7.93	2.78	K to Li, Na, Cs (Marcia and Pyle, 2012)	distortion of active site, splicing defect
BamHI (*B. amyloliquefaciens*)	2BAM	2.00	3.1.21.4	Lys61	5.87	via Tyr65 (3.08 Å)	computational electrostatic model (Sun et al., 2003)	NA	Lys126	6.23	2.90	computational electrostatic model (Sun et al., 2003)	NA
Exo-λ (*Escherichia virus lambda*)	4WUZ/3SM4	2.38/1.88	3.1.11.3	Lys131	4.44	2.97	K131A (Zhang et al., 2011)	no exonuclease activity	Arg28	9.55	2.96	R28A (Zhang et al., 2011)	no exonuclease activity
Cas9 (*S. pyogenes*)	4CMQ/5F9R	3.09/3.40	3.1.–.–	Lys974	6.05	3.08	K974A (Zhang et al., 2016)	altered substrate binding and kinetics	Lys968	8.82	3.79	K968A (Zhang et al., 2016)	altered substrate binding and kinetics
DNA Pol-η (*H. sapiens*)	4ECS	1.95	2.7.7.7	Lys231	3.98	2.68	K279A in yeast (Johnson et al., 2003)^d^	decreased efficiency	Lys224	4.71	3.43	NA	NA
FluA, endonuclease PA subunit (influenza A virus)	2W69	2.05	2.7.7.48	His41	2.91	3.47	NA		Arg84	6.74	x^e^	NA	
				Lys134	4.17	2.77	K134A (Crepin et al., 2010)	reduced thermal stability and no activity	Lys137	13.33	x^e^	K137A (Crepin et al., 2010)	decreased activity

The complete list of enzymes analyzed in this work is reported in Tables S1 and S2.

a Closest distance between the ion/amino acid corresponding to K1 or K2 and the M_A_-M_B_ center.

b Closest distance between the ion/amino acid corresponding to K1 and the acidic residues that chelate M_A_-M_B_.

c Closest distance between the ion/amino acid corresponding to K2 and the substrate.

d See Table S1.

e No substrate in the active site; NA, not available.

Over the years, 3D structures have revealed additional important catalytic elements in specific classes of two-metal-ion enzymes. For example, in DNA polymerases a third transiently bound divalent cation and a positively charged, highly flexible residue (Arg61 in the human DNA polymerase-η [Pol-η]) facilitate product release (Gao and Yang, 2016, Genna et al., 2016a, Nakamura et al., 2012). However, so far no catalytic element other than M_A_-M_B_ and their coordinating acidic residues appeared conserved across different classes of two-metal-ion enzymes.

**Figure 1:**
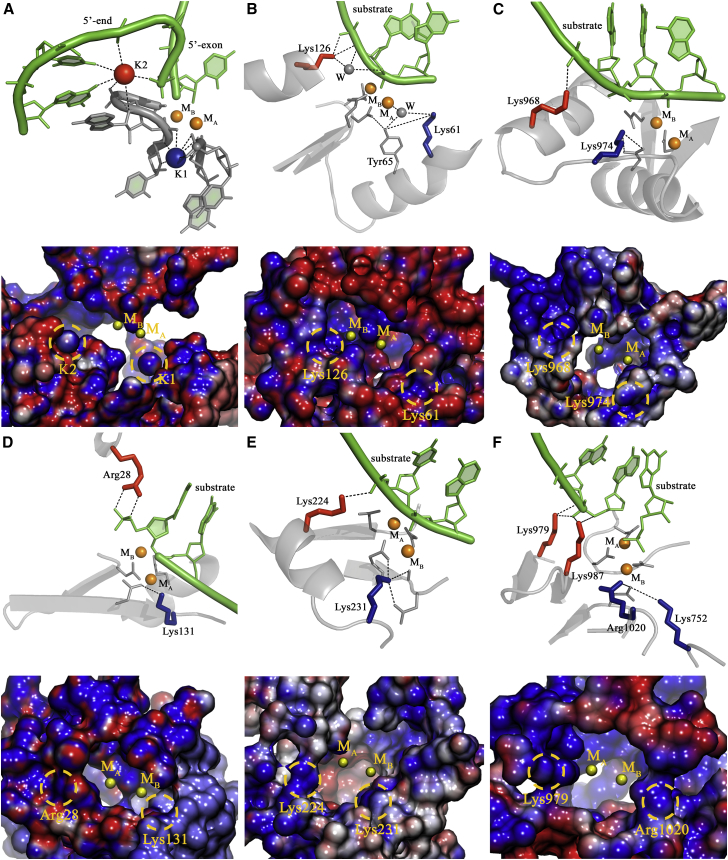
Structural Comparison of the Catalytic Sites of Representative Two-Metal-Ion Enzymes (A) Group II intron (PDB: 4FAR). (B) Restriction endonuclease BamHI (PDB: 2BAM). (C) SpyCas9 (RuvC active site modeled from PDB: 4CMQ and 5F9R). (D) Exonuclease Exo-λ (modeled from PDB: 4WUZ and 3SM4). (E) DNA polymerase Pol-η (PDB: 4ECS). (F) RNA polymerase Pol-II (PDB: 2E2H, predicted from medium-resolution structures, see Table S2). Each top panel presents the structure of the active site of the respective molecules in ribbon-stick representation. End each bottom panel presents the molecular surface surrounding the two-metal-ion active sites (radii 15 Å) colored by intensity of electrostatic potential (−10 kT/[darkest red] to +10 kT/e [darkest blue]). Catalytic divalent ions (M_A_-M_B_) are depicted as orange spheres, substrates are represented in green, and the backbone of the corresponding macromolecule is in gray, with acidic residues coordinating M_A_-M_B_ represented as sticks. K1- and K2-like elements are depicted as spheres (for ions) or sticks (for amino acids) in blue and in red, respectively. “W” indicates water molecules. Black dashed lines indicate ionic or hydrogen-bond interactions established by K1-and K2-like elements with active site residues and reactants. K1-like elements rigidify the residues that coordinate M_A_-M_B_, while K2-like elements orient the substrates into the active site. All structures have been drawn using PyMOL.

**Figure 2:**
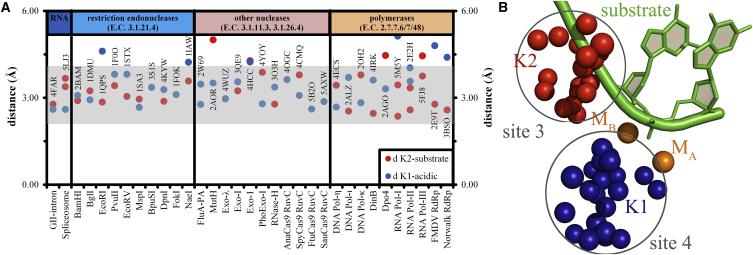
Positioning of K1- and K2-like Elements with Respect to the Two-Metal-Centered Active Site (A) The plot reports distances in angstroms of K1-like elements from the acidic residues that coordinate M_A_-M_B_ (d K1-acidic, blue dots) and of K2-like elements from the substrate (d K2-substrate, red dots). The gray shade covers the optimal range of distances for hydrogen bonds and ionic interactions (Ippolito et al., 1990). Several outliers correspond to structures solved at low resolution (i.e., PDB: 5FJ8) or with no metals in the active site (i.e., PDB: 1IAW). For enzymes in which K1 contacts acidic residues indirectly (PDB: 2BAM, 1DMU, 1QPS, and 2ALZ), we reported the closest distance to the linking residue (see also Tables 1 and S1). For structures where K2 is present, but the substrate is not resolved in the PDB file (PDB: 3S1S, 1FOK, 4OGC, 5B2O, and 5AXW), we did not plot any data point (see Tables 1 and S1). In exo-λ structure PDB: 4WUZ, the “d K1-acidic” and “d K2-substrate” data points overlap and only the blue dot is actually visible. The names of the enzymes are on the x axis, corresponding PDB codes are indicated on top of every data point. Enzymes are arranged by classes and their respective Enzymatic Classification (E.C.) numbers are indicated on the top of the graph. (B) Overlap of the structures reported in (A), aligned manually in Coot using the substrate and the two-metal-ion center as a guide. For clarity, substrate (green cartoon) and M_A_-M_B_ center (orange spheres) are represented only for endonuclease BamHI. Blue and red spheres represent the position of the K1- and K2-like elements from all structures, respectively. For clarity, we only represent the position of the potassium ion (for ribozymes), of the epsilon nitrogen atom (for lysine residues), or of the guanidinium carbon atom (for arginine residues), as appropriate. Gray circles represent spheres of a radius of 4.0 Å (K1) and 3.5 Å (K2) and identify sites 4 (K1) and 3 (K2), respectively, as described in Figure 4 and in the main text.

In this respect, recent crystal structures of self-splicing group II intron ribozymes revealed that, besides M_A_-M_B_ and surrounding conserved nucleotides, two potassium ions (K1 and K2) located in the vicinity of the active site are essential for catalysis (Figures 1A and 2; Table 1) (Marcia et al., 2013a, Marcia et al., 2013b, Marcia and Pyle, 2012, Marcia and Pyle, 2014). Interestingly, the active site of this RNA enzyme is strikingly similar to that of the protein enzyme BamHI, a type II restriction endonuclease, despite their fundamentally different biopolymer scaffold (Marcia and Pyle, 2012). This surprising structural similarity at the active site suggested the possible existence of enzymatic amino acid counterparts of K1 and K2 in BamHI (Marcia and Pyle, 2012). The presence of K1- and K2-like amino acids in protein enzymes that process DNA and RNA would infer a more extended set of functional components for the enzymatic processing of nucleic acids through the two-metal-ion mechanism.

Intrigued by this hypothesis, here we examined a broad set of DNA/RNA processing enzymes, comprising splicing machineries such as the group II intron and the spliceosome, nucleases such as the contemporary Cas9 genome-editing tool, and DNA and RNA polymerases. Remarkably, in all these enzymes we identified two positively charged residues with a conserved spatial localization in the second coordination shell of the two-metal-aided catalytic site similar to K1 and K2 in the group II intron ribozyme. These structural elements define a larger two-metal-ion-centered enzymatic structure, which potentially reflects a common strategy of multiple two-metal-ion enzyme classes to ensure fidelity, substrate specificity, and catalytic efficiency for DNA and RNA processing.

## Results

### Second-Shell Positively Charged Residues Define Geometry and Electrostatic Potential of the Active Site of Two-Metal-Ion Enzymes

In the group II intron, potassium ion K1 anchors the residues that coordinate M_A_-M_B_, while potassium ion K2 stabilizes the 5′-splice junction before catalysis and the scissile phosphate after catalysis (Marcia and Pyle, 2012, Marcia and Pyle, 2014, Marcia et al., 2013b). K1 and K2 produce two discrete regions of positive electrostatic potential (+7.4 kT/e) at 4–8 Å from the active site, which interrupts the negative potential created by first-shell coordinators of M_A_-M_B_ (Figure 1A). Replacement of K1 with smaller (i.e., lithium and sodium) or bigger (i.e., cesium) ions prevents M_A_-M_B_ binding, thus affecting catalysis (Marcia and Pyle, 2012). In summary, the K1 and K2 ions rigidify the architecture of the M_A_-M_B_ binding pocket, mediate functional conformational changes during the splicing cycle, and stabilize reactants in the pre- and post-catalytic states (Marcia and Pyle, 2012).

Stiffening of the active site, modulation of its electrostatic environment, and orientation of the reactants relative to the M_A_-M_B_ center are needed by two-metal-ion enzymes to ensure specificity in substrate recognition and to augment the fidelity of the reaction (Hanoian et al., 2015, Jeltsch et al., 1993, Kurpiewski et al., 2004, Ramachandrakurup et al., 2016, Warshel et al., 2006). Therefore, we reasoned that counterparts of K1 that rigidify the first coordination shell of M_A_-M_B_ and counterparts of K2 that help orienting the substrates into the active site may exist in other families of two-metal-ion enzymes.

We thus selected two-metal-ion enzymes belonging to six different enzymatic classes, chosen so that there are high-resolution (≤3 Å) structures in the PDB having an integer metal ion center for at least one enzyme of every considered class (Table 1). This selection resulted in a collection of 49 3D structures, which we systematically compared to identify putative K1 and K2-like residues in their active sites (Figure 2; Table S1).

### Functional Monovalent Cations of the Group II Intron Match Functional Basic Amino Acids of Endo- and Exonucleases, Including Cas9

We first superposed the coordinates of *Oceanobacillus iheyensis* group II intron with those of substrate-bound BamHI. Two BamHI lysines match the group II intron K1 and K2 ions (Figure 1B). Specifically, Lys61 engages in a hydrogen-bond network with catalytic Asp94 via Tyr65 (Marcia and Pyle, 2012), resembling K1, and Lys126 forms a direct contact with the substrate DNA, analogously to K2 (Table 1). Lys61 and Lys126 generate an electrostatic potential distribution around the active site, analogous to that observed for K1 and K2 ions in the group II intron (+8.5 kT/e; Figure 1B). Notably, Lys61 and Lys126 are strictly conserved in different bacterial species (Data S1) and aid cognate DNA binding via large amplitude motions (Uyar et al., 2011) and favorable Coulombic interactions (Sun et al., 2003) (Table 1).

Interestingly, a similar structural architecture with positively charged residues arranged in the second coordination shell of M_A_-M_B_ can also be observed: (1) in other orthodox PD-(D/E)XK restriction endonucleases (Pingoud and Jeltsch, 2001), such as EcoRI, which produces 5′ overhangs, BglI, which produces 3′ overhangs, and PvuII, which produces blunt ends (Data S1; Figure S1; Table S1); and (2) in other subfamilies of bacterial restriction endonucleases, such as class IIP endonuclease MspI, class IIG endonuclease BpuSI, class IIM endonuclease DpnI, class IIS endonuclease FokI, and class IIE endonuclease NaeI (Table S1). Such second-shell positively charged residues are highly conserved for all these enzymes (Data S1) and participate in DNA binding and catalysis (Jeltsch et al., 1993, Kurpiewski et al., 2004, Ramachandrakurup et al., 2016) (Table S1). Other bacterial endonucleases, such as MutH, which is involved in DNA repair (Pingoud and Jeltsch, 2001), or viral endonucleases, such as the PA subunit of influenza virus RNA polymerases (FluA, FluB, FluC), which is involved in cap-snatching (Dias et al., 2009), also present evolutionarily conserved residues that impair catalysis if mutated at K1- and K2-like positions nearby their two-metal-ion active sites (Data S1; Figure S1; Table S1).

Second, we identified K1- and K2-like residues in the RuvC domain of Cas9, an RNA-guided DNA bacterial endonuclease associated with the CRISPR type II adaptive immunity system (Jiang and Doudna, 2015) and nowadays largely exploited for genome editing (Wright et al., 2016). A complete picture of the RuvC active site of Cas9 can be obtained by superposition of a *Streptococcus pyogenes* Cas9 (SpyCas9) structure that displays the M_A_-M_B_ center, but no substrates (PDB: 4CMQ), with a SpyCas9 structure that displays the substrate DNA but no divalent metals (PDB: 5F9R) (Jinek et al., 2014). Such superposition suggests that Lys974 and Lys968 occupy analogous structural locations as the K1 and the K2 ions of the group II intron, respectively (Figure 1C). Lys974 and Lys968 also produce two regions of positive electrostatic potential on the surface of SpyCas9 (+9.8 kT/e and +13.0 kT/e, respectively) discontinuing the negative potential (−9.5 kT/e) that surrounds the catalytic metals (Figure 1C). Interestingly, Lys974 and Lys968 have precise counterparts in the Cas9 active site of *Actinomyces naeslundii* (AnaCas9), *Francisella tularensis* (FtuCas9), and *Staphylococcus aureus* (SauCas9), and in RNase-H, which is evolutionarily related to the RuvC domain of Cas9 (De Vivo et al., 2008, Ho et al., 2010, Jiang and Doudna, 2015, Jinek et al., 2014) (Figure S1; Data S1).

Finally, K1- and K2-like residues are also persistently conserved among exonucleases. *Escherichia* virus lambda exonuclease (Exo-λ), which catalyzes 5′ to 3′ exonucleolytic cleavage in dsDNA (Pingoud and Jeltsch, 2001), presents Lys131 in a position analogous to the K1 ion and Arg28 in a position analogous to K2 (Figure 1D). Both residues are strictly conserved (Data S1), abolish catalysis if mutated (Zhang et al., 2011) (Table 1), and have parallel counterparts in LHK-exonuclease and RecE (Yang et al., 2011). Counterparts of Lys131 and Arg28 in human exonuclease-I (Exo-I), which catalyzes 3′ to 5′ exonucleolytic cleavage in dsDNA, are Lys85 and Arg92, respectively. Intriguingly, Arg92 forms a bifurcated H-bond interaction with both scissile and 5′ end phosphate groups, as observed for K2 ions in group II introns. Finally, similar structural determinants are also present in prokaryotic exonucleases, such as Exo-1 of the mesophilic host *Escherichia coli* (conserved His181 and Arg165 in K1- and K2-like positions, respectively) and Exo-I of the extremophilic host *Pyrococcus horikoshii* (PhoExo-I, Arg142, and Lys136 in K1- and K2-like positions, respectively).

### K1/K2-like Residues Are Also Similarly Located Nearby the Catalytic Site of DNA and RNA Polymerases

Having assessed that K1- and K2-like residues are recurrent in enzymes that catalyze scission of phosphodiester bonds, we then analyzed enzymes that catalyze the synthesis of phosphodiester bonds, such as DNA and RNA polymerases. Prominent classes of DNA and RNA polymerases, which satisfy our selection criteria (3D structures available at a resolution higher than 3 Å and displaying an integer metal ion center, see above), are viral RNA-directed RNA polymerases (RdRps) (Baltimore, 1971) and Y-family DNA polymerases, which are involved in DNA repair (Genna et al., 2016a, Genna et al., 2016b, McCulloch et al., 2004, Nakamura et al., 2012, Patra et al., 2015).

We first considered viral RdRps. Norwalk virus RNA polymerase presents Lys374 in a position equivalent to K1 and Arg392 in a position analogous to K2 (Figure S1). Structure-based sequence alignments suggest that Lys374 and Arg392 are highly conserved in RdRps from *Caliciviridae* and from other single-stranded positive RNA viruses (viral group IV, according to the Baltimore classification [Baltimore, 1971]), i.e., *Picornaviridae* (Data S1) and *Flaviviridae* (Table S1). In other viral groups, the presence of such residues is less evident from sequence alignments, but structural superposition helps identifying putative K1- and K2-like counterparts. For instance, in the RdRps from *Orthomixoviridae* and *Bunyaviridae*, the role of K1 and K2 may be played by residues consistently located in ultraconserved polymerase motifs D, F, and G, which are common to all single-stranded negative RNA viruses (viral group V) and to the reverse transcriptase of retroviruses (viral group VI; Table S1) (Gerlach et al., 2015, Muller et al., 1994).

Second, we analyzed the human Y-family DNA Pol-η. Here, Lys231 anchors M_A_-M_B_ in a position analogous to the K1 ion, while Lys224 orients the phosphate backbone in the proximity of the cleavage site in a position analogous to K2 (Figure 1E). Both Lys231 and Lys224 are strictly conserved (Data S1) and are essential for substrate binding and stabilization (Ummat et al., 2012) (Table 1). For instance, Lys231 in the pre-reactive complex contributes to properly position the incoming substrate aiding catalysis, while in the post-reactive state it counterbalances the negative charge of departing pyrophosphate (Genna et al., 2016a). The surface electrostatic map of Pol-η confirms that Lys231 and Lys224 produce discrete regions of positive electrostatic potential at 8–9 Å from the catalytic metals, mimicking the electrostatic effect of K1- and K2-like residues in endo- and exonucleases. Moreover, Lys231 and Lys224 have precise counterparts in other human Y-family DNA polymerases, such as DNA polymerase-ι (Pol-ι) and DNA polymerase-κ (Pol-κ), in yeast DNA polymerases, such as Pol-η from *S. cerevisiae*, and in mesophilic and thermophilic bacterial DNA polymerases, such as *E. coli* DinB and *S. solfataricus* Dbh and DPO4 (Table S1; Figure S2) (Uljon et al., 2004). Human and yeast Pol-η, human Pol-ι, human Pol-κ, *S. solfataricus* Dpo4, and *E. coli* DinB represent a particularly interesting subset of enzymes within our analysis, because all these enzymes share a common ancestor but have diverged over one billion years in evolution. Moreover, all their 3D structures are available at high resolution and in a catalytically active conformation, displaying an integer active site and substrate. Thus, this subset of two-metal-ion enzymes represents a curated dataset to analyze the conservation in structural distribution of K1- and K2-like residues around the two-metal ion active site. A superposition of these structures remarkably shows that both K1- and K2-like residues localize within 1.5-Å-radius spheres at about 4.5 Å from the two-metal-ion center (Figure S2), thus suggesting that their structural position has been preserved over evolution.

**Figure 3:**
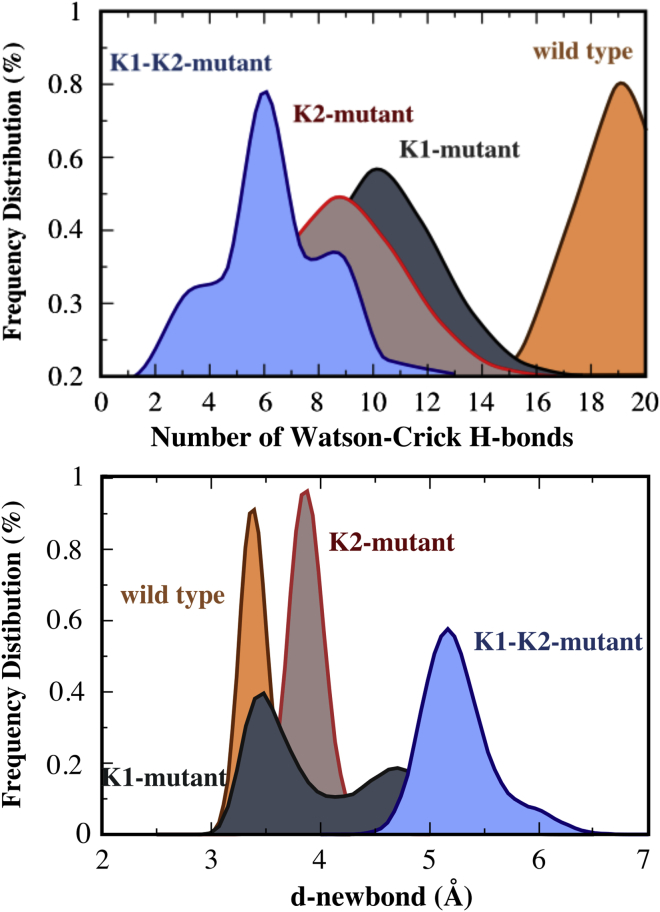
Molecular Dynamics Simulations of DNA Polymerase Pol-η Mutated at the K1- and K2-Sites Top: frequency distribution of the Watson-Crick inter-strand H-bonds in wild-type Pol-η, and in Pol-η mutated at the K1 site (K231A, K1-mutant), at the K2 site (K224A, K2-mutant), and at both K1 and K2 sites (K224A/K231A, K1-K2-mutant). Bottom: frequency distribution of the length *d-newbond*, defined in the main text (Genna et al., 2016a).

We further assessed the structural role of K1- and K2-like elements in the active site through extensive (∼1.5 μs) classical molecular dynamics (MD) simulations of human wild-type DNA Pol-η in complex with the substrate dsDNA and the incoming dATP, and of three Pol-η mutants derived from such a wild-type system (K231A, K224A, and K224A/K231A mutants, see the STAR Methods). We found that, along our MD simulations, both K231A and K224A mutations lead to displacement of the substrate dsDNA, to a lower number of inter-strand Watson-Crick H-bonds in the dsDNA, and to amplified fluctuations of *d-newbond*, i.e., the distance between the two reactive groups, the 3′-O^−^ of the terminal base of the DNA and the α-phosphorus of dATP (Genna et al., 2016a) (Figures 3 and S3). In the Pol-η K231A mutant, the dsDNA reached a root-mean-square deviation (RMSD) value of 2.5 ± 0.4 Å, which is ∼1 Å higher with respect to the wild-type form. Concomitantly, the number of the Watson-Crick inter-strand H-bonds dropped to 11 from the optimal value of 20 observed in the wild-type system (Figure 3). This distortion, which indicates partial dsDNA unfolding, causes the disruption of the Michaelis-Menten complex, as indicated also by fluctuations of *d-newbond*, which reached an average value of 4.0 ± 0.6 (i.e., ∼0.7 Å longer if compared with the wild-type system, where *d-newbond* is 3.3 Å [Genna et al., 2016a]). Also, in the Pol-η K224A mutant, we observed similar conformational changes. The dsDNA reached an RMSD value of 3.8 ± 0.3 Å and a reduced number of nine Watson-Crick inter-strand H-bonds. The *d-newbond* length was 3.8 ± 0.1 Å, which suggests a conformation likely less prone to react (Figures 3 and S3). Finally, the most severe distortions were observed in the Pol-η K224A/K231A double mutant, where the dsDNA substrate reached an RMSD value of 3.8 ± 0.8 Å, the inter-strand H-bonds dropped to 6, and *d-newbond* reached an average value of 5.5 ± 0.4 Å after only ∼300 ns (Figures 3 and S3). Taken together, these data indicate significant distortions of the dsDNA upon mutation of K1- and/or K2-like elements.

### Common Characteristics of K1- and K2-Counterparts in Nucleic Acid-Processing Enzymes

Our work suggests that K1- and K2-like residues are a common feature of nucleases and polymerases. Some K1- and K2-like residues play idiosyncratic roles in specific enzyme classes. For instance, K1-like Lys144 of BglI and Lys131 of Exo-λ activate the reaction nucleophile (Yang et al., 2011), while K2-like Arg61 of Pol-η facilitates pyrophosphate departure after catalysis (Genna et al., 2016a). However, we noted that all K1- and K2-like residues found in the classes of two-metal-ion enzymes considered here share the following characteristics.

**Figure 4:**
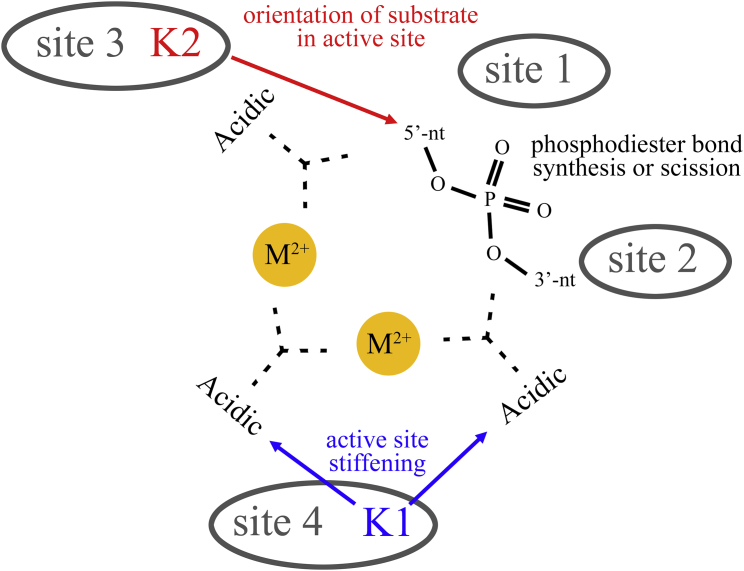
Structural Model Recapitulating Positions and Possible Roles of K1- and K2-like Positive Charges in the Active Site of Two-Metal-Ion Enzymes In the active site, strictly conserved acidic residues (“acidic”) coordinate two divalent ions (M^2+^) that catalyze synthesis or scission of phosphodiester bonds in the DNA/RNA substrate. “5′-nt” and “3′-nt” indicate nucleotides on the 5′ and 3′ side of the reaction site, respectively. In all enzymes discussed in this work, the active site is completed by the presence of two positively charged elements that correspond to the group II intron K1 and K2 ions. These structural elements are either basic amino acids or monovalent cations. These elements likely contribute to rigidify the active site for optimal M^2+^ binding and to orient the substrate for catalysis. Sites 1, 2, and 3 are structural positions previously identified by Steitz and Steitz (1993), and site 4 is a structural site identified as described in the main text (see the Results section). Sites 3 and 4 encompass K2- and K1-like residues, respectively.

First, we show that they occupy analogous structural positions in the second coordination shell of the active site. A superposition of all structures analyzed in our work provides a quantitative assessment of the level of structural conservation of K1- and K2-like elements. All K1-like residues localize within a 4.0-Å-radius sphere at about 6.0 Å from the M_A_-M_B_ center, and all K2-like residues localize within a 3.5-Å-radius sphere at about 8.0 Å from the M_A_-M_B_ center (Figure 2). In particular, the K2-like residue forms ionic or hydrogen-bonding interactions with the reaction substrate and/or product (Figure 2; Tables 1 and S1). Within the typical active site geometry of two-metal-ion enzymes (Steitz and Steitz, 1993), K2-like thus occupies “site 3” (Figure 4). Instead, the K1-like residue forms ionic or hydrogen-bonding interactions with residues of the first coordination shell of M_A_-M_B_ (Figure 2; Tables 1 and S1). As such, the K1-like residue occupies a site hereafter named “site 4.” In this expanded two-metal-ion-centered architecture, site 4 is structurally juxtaposed to sites 1 and 2, which flank the scissile phosphate on the 5′- and the 3′-sides, respectively, and it is typically located closer to the 3′ end side of the substrate and thus closer to site 2 than to site 1. Relative to the substrate backbone, site 4 is consistently located opposite the nitrogenous base moieties (Figure 4).

Second, as expected for such basic residues, we found that K1- and K2-like analogs produce discrete peaks of second-shell-positive electrostatic potential, which discontinues in a similar manner the negative potential surrounding M_A_-M_B_ (Figure 1). Finally, we demonstrate that K1- and K2-like residues are highly conserved for each enzymatic class (Data S1), and we report evidence from available literature, suggesting that their mutations are associated to functional defects across the entire dataset (Tables 1 and S1).

## Discussion

Here, we have analyzed six classes of RNA and protein enzymes that follow a two-metal-ion mechanism of catalysis, including self-splicing group II introns, nucleases, and DNA/RNA polymerases. In our work, we have specifically considered high-resolution structures (<3 Å) that display all reactants and metal ions in the enzymatic active site. By multiple-sequence and structural alignments, molecular modeling, MD simulations, and electrostatic potential maps, we have identified two second-shell and positively charged elements (basic amino acids or monovalent cations) that are located in the vicinity of the two-metal-aided active site. In all the analyzed structures, despite the different size, shape, composition, and biological function of the respective enzymes, we have found that these elements share four common characteristics: they occupy analogous structural positions, they help modulating both the electrostatics in the surroundings of the active site and substrate stability, they are evolutionarily conserved, and they are associated to functional defects if mutated (see Tables 1, S1, and S2). These observations and results allow distinguishing K1- and K2-like residues from any other basic amino acid present in the proximity of two-metal-centered active sites.

Also, these common properties of the K1- and K2-like residues can serve as a guide to identify analogous residues in other classes of two-metal-ion enzymes, particularly those for which structures can be determined only at medium-low resolution (>3 Å). To exemplify the predictive potential of our analysis of K1- and K2-analogs, we have analyzed the recent 3.9–4.0 Å resolution X-ray and cryoelectron microscopy structures of DNA-directed RNA polymerases (DdRp), which synthetize RNA in eukaryotic cells (Cramer et al., 2001, Tafur et al., 2016), and the 3.6–5.8 Å cryoelectron microscopy structures of the spliceosome, a mega-Dalton machinery which catalyzes nuclear pre-mRNA splicing in eukaryotes and which is evolutionarily linked to the group II intron (Fica et al., 2014, Pyle and Lambowitz, 2006, Sharp, 1991). In these enzymes, we can detect specific basic residues at the putative K1 and K2 sites. In those sites where two basic residues could potentially act as K1 (or K2) analogs, we are presenting both alternatives below (Table S2). Such ambiguity likely derives from the fact that the DdRps and spliceosome structures are still solved at only medium resolution, that ions in the M_A_-M_B_ center are missing in some structures (i.e., RNA polymerase I [Pol-I] and RNA polymerase III [Pol-III]), and that enzymatic assays on mutants are not available yet.

**Figure 5:**
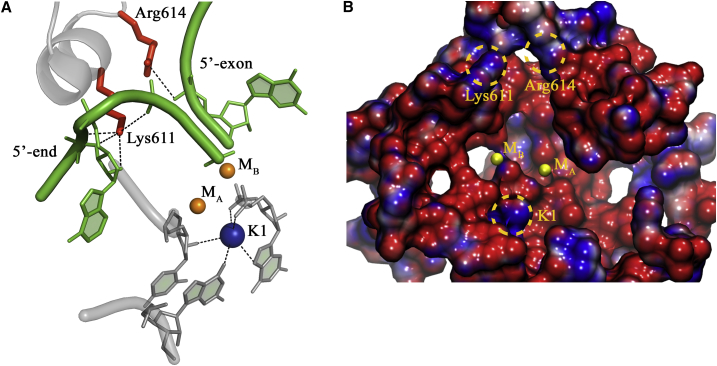
Prediction of K1- and K2-like Residues in Medium-Resolution Cryoelectron microscopy Structures of the Spliceosome (A) Representation of the active site of the spliceosomal C complex from *S. cerevisiae* immediately after branching (PDB: 5LJ3). (B) Electrostatic distribution around the active site. Structural elements are oriented and color coded as in Figure 1. A putative potassium ion at the K1-site is indicated (K1), although not modeled in the actual structure (see main text for details).

We suggest that in *S. cerevisiae* RNA polymerase II (Pol-II) (Cramer et al., 2001), Lys752 (Rbp1 subunit), and/or Arg1020 (Rbp2) occupy the K1 position, and Lys979 and/or Lys987 (Rbp2) occupy the K2 position (Figure 1F). Mutation of these amino acids is lethal in yeast (Table S2). Moreover, these amino acids are strictly conserved across Pol-II from yeast to humans, and they are also conserved in RNA Pol-I and Pol-III (Tafur et al., 2016) (Data S1). Instead, in the spliceosome, we noticed that a putative K1 cavity essentially identical to the one of group II intron exists between U6-snRNA residues G52, A59, G60, and U80 (Figure 5; Table S2). Although not modeled in the current structures, a potassium ion could optimally fit this putative K1 site at least at certain steps of the catalytic cycle, which would explain the spliceosomal dependence on potassium for splicing and debranching (Hardy et al., 1984, Tseng and Cheng, 2013). In addition, Lys611 (PROCN domain of the Prp8 subunit [Staub et al., 2004], Data S1), which stabilizes the curvature of the intron backbone (U2 and U4) proximal to the splicing junction in the C complex, and/or conserved Arg614 (Prp8), which makes contacts with the exon in the C^∗^ complex (Figures 2 and 5; Table S2), seem to constitute optimal spliceosomal counterparts of K2. These residues produce regions of positive electrostatic potential at 7–9 Å from the active site (+6.7 kT/e), similar to the K2 ion in group II intron (Figure 5).

Notably, our analysis also spotted enzymatic classes that do not possess K1- and/or K2-like residues. For instance, class IIF endonucleases present K2 counterparts (i.e., Arg102 in NgoMIV, Arg218 in SfiI, and Arg152 in SgrAI, respectively), but no K1 counterparts, the role of the latter possibly being replaced by conserved lysines that directly coordinate M_A_-M_B_ (i.e., Lys187 in NgoMIV, Lys102 in SfiI, and Lys242 in SgrAI) or by other non-basic residues (Table S1). DNA polymerase β (Pol-β) also represents an exception, because in this enzyme the M_A_-M_B_-binding acidic residues are not coordinated by residues near the putative K1-site (Arg183 or Arg149), but rather by the K2-like residue (Arg254; Table S1). A third notable case is represented by LAGLIDADG homing endonuclease I-DmoI, an enzyme that performs two DNA hydrolysis reactions using two asymmetric active sites and three divalent metal ions (M_A_, M_B_, and M_C_). In the first and faster reaction, the non-coding DNA strand is cleaved with support of M_A_ and M_C_, while in the second and slower reaction, the coding DNA strand is cleaved with support of M_B_ and M_C_. Our analysis shows that functionally crucial Lys120 (Molina et al., 2015) occupies the K1-like position and Lys130 the K2-like position of the first active site (Table S1). Instead, in the second active site, only a K2-like residue (Lys 43), but no K1-like residue could be identified (Table S1). The asymmetry of the two active sites of I-DmoI, and their consequently different catalytic efficiencies (Molina et al., 2015), is thus perfectly mirrored by a different architecture of the K1 and K2 positions, with only the most efficient active site presenting both K1 and K2 positions occupied by a basic amino acid. These considerations suggest that K1- and K2-like residues have been specifically selected by diverse, yet not all, two-metal-ion enzyme classes for DNA/RNA processing. Further analysis is thus needed now to clarify why certain two-metal-ion enzymes preserved K1- and K2-like residues, and elucidate their exact catalytic role for DNA and RNA processing.

Preserved spatial localization of K1- and K2-like residues and catalytic defects caused by their mutation further suggest that the somewhat obvious presence of such positively charged residues proximal to the anionic nucleic acid backbone may actually be coupled to a functional role (Tables 1 and S1). Possibly, K1- and K2-like residues affect DNA and RNA processing reactions directly, by participating in the chemical or physical steps of catalysis. For instance, in group II introns K1 and K2 mediate conformational changes, such as the transition from the first to the second step of splicing (Marcia and Pyle, 2012), and in polymerases these residues help translocation and nucleotide addition (Genna et al., 2016b, Wu et al., 2014, Zhao et al., 2013). Alternatively, K1 and K2 may play indirect functional roles, by contributing to stabilize the overall architecture of the active site. For example, these residues may help rigidifying the coordination center of metals M_A_-M_B_, while also correctly orienting the substrates into the active site, as suggested by our MD simulations (Figures 3 and S3). Future validation is clearly required to confirm or disprove such mechanistic hypotheses. In this respect, future mutagenesis and engineering studies on K1- and K2-like residues may have prospective applicability in designing enzymes with improved substrate selectivity or higher substrate specificity, which would have immediate biotechnological impact, e.g., in the optimization of CRISPR-Cas9 genome-editing machineries. Furthermore, design of small molecules that bind to K1- and K2-like sites may lead to identify antimicrobial or antitumoral drugs with improved specificity against two-metal-ion enzymes (Berdis, 2008). Finally, reproducing the chemical architecture of the K1-K2-M_A_-M_B_ phosphorus-oxide cluster formed by the group II intron may lead to develop nucleic acid-processing biocatalysts, such as DNAzymes or RNAzymes with important applications in nanoengineering (Li et al., 2011). We therefore hope that our structural observations will now stimulate further studies to clarify possible functional implications of K1- and K2-like residues in two-metal-ion enzymes for DNA and RNA processing.

## STAR★Methods

### Key Resources Table

**Table:** 

REAGENT or RESOURCE	SOURCE	IDENTIFIER
**Software and Algorithms**		

BLAST	Altschul et al., 1990	https://blast.ncbi.nlm.nih.gov/Blast.cgi
ClustalOmega	Sievers et al., 2011	http://www.ebi.ac.uk/Tools/msa/clustalo/
T-COFFEE	Notredame et al., 2000	http://tcoffee.crg.cat/
COOT	Emsley and Cowtan, 2004	https://www2.mrc-lmb.cam.ac.uk/personal/pemsley/coot/
PyMOL	Schrödinger, 2010	https://pymol.org/
APBS	Baker et al., 2001	http://www.poissonboltzmann.org/
AMBER	Wang et al., 2004	http://ambermd.org/

### Contact for Reagent and Resource Sharing

Further information and requests for resources and reagents should be directed to and will be fulfilled by the Lead Contact, Marco Marcia (mmarcia@embl.fr).

### Method Details

#### Sequence and Structural Alignments

Representative sequences of each enzyme have been selected in BLAST (Altschul et al., 1990) and multiple-sequence alignments have been performed in ClustalOmega (Sievers et al., 2011). Structure-based sequence alignments have been performed using T-COFFEE (Notredame et al., 2000), as described previously (Marcia et al., 2010).

Structural alignments have been performed manually in Coot (Emsley and Cowtan, 2004) using the di-nuclear metal center M_A_-M_B_, the substrates, and/or the coordinating acidic residues in the first resolution shell of M_A_-M_B_ as a guide. The figures depicting the structures were drawn using PyMOL (Schrödinger, 2010).

#### Calculations of Electrostatic Potential Maps

Calculations of the electrostatic molecular surfaces were performed by solving a nonlinear Poisson-Boltzman equation with APBS (Baker et al., 2001) as described previously (Kazantsev et al., 2009). For all the systems, we used a multilevel grid approach with a fixed grid length of 129 points in each spatial direction obtaining a grid spacing of ≤ 0.365 Å. To generate the electrostatic maps, all biomolecules were treated with a low dielectric medium (ɛ = 2) (Misra and Draper, 2001) and the surrounding solvent as a high dielectric continuum (ɛ = 78.54). The ionic strength was set to 150 mM corresponding to a Debye length of 8 Å, while temperature was set to 300 K. AMBER package (Wang et al., 2004) was used to add hydrogen atoms to crystallographic models and the AMBER ff99SB-ILDN force field (Lindorff-Larsen et al., 2010) was used to derive atomic radii and charges. In order to diminish the sensitivity of computations to the grid setup, we have used cubic B-spline discretization method to map the charges of our systems into the grid points and the nine-point harmonic averaging approach to the surface-based dielectric and ion-accessibility coefficients. Finally, we applied Dirichlet boundary conditions by using multiple Debye–Huckel functionality.

#### Molecular Dynamics Simulations

##### Structural Models

We considered four different systems: human wild type DNA Pol-η [PDB id.: 4ECS, (Genna et al., 2016a)], and human DNA Pol-η mutated at the K1 site (K231A), at the K2 site (K224A), and at both K1 and K2 sites (K224A/K231A). All the systems are a ternary complex where human DNA Pol-η is bound to the dsDNA substrate and the incoming dATP.

##### Molecular Dynamics Simulation Set Up

The all-atom AMBER/parm99SB-ILDN (Lindorff-Larsen et al., 2010) force field was adopted for the Pol-η in complex with dsDNA, whereas dATP was treated with the general amber force field (Wang et al., 2004). The atomic charges were derived by fitting the electrostatic potential according to the Merz-Singh-Kollman scheme (Besler et al., 1990) also known as the RESP fitting procedure (Table S3). The length of all covalent bonds, including hydrogen atoms, was set using the LINCS algorithm, allowing a time-integration step of 2 fs. All simulations were performed using Gromacs 4.6.1 code (Berendsen et al., 1995). Long-range electrostatic interactions were calculated with the particle mesh Ewald method with a real space cutoff of 12 Å. Periodic boundary conditions in the three directions of Cartesian space were applied. Constant temperature (310 K) was imposed using Langevin dynamics (Grest and Kremer, 1986) with a damping coefficient of 1 ps. A constant pressure of 1 atm was maintained with Langevin-Piston dynamics with a 200 fs decay period and a 50 fs time constant. The metal active site was treated with a flexible non-bonded approach based on the ‘atoms in molecules’ partitioning scheme of the DFT-BLYP electronic density of the active site [Table S3, (Dal Peraro et al., 2007)]. We could thus consider the charge-transfer interactions between Mg^2+^ ions and their ligands, permitting possible structural rearrangements at the active site during the MD simulations. All the simulated systems were hydrated using TIP3P (Jorgensen et al., 1983) water molecules. A total of 7 Mg^2+^ ions were added to each system to reach a final concentration of ∼1 mM, while Na^+^ and Cl^−^ ions were added to neutralize the total charge. The size of the final systems was approximately 115 × 95 × 93 Å^3^, with ∼35,000 water molecules, resulting in a total number of ∼102,000 atoms each.

We adopted the following simulation protocol: the systems were minimized using a steepest-descent algorithm and then slowly heated up to 310 K in 10 ns for a total of 2000 steps. The first 50 ns of production run are considered as the equilibration phase. Approximately ∼500 ns of MD simulations were collected in the NPT ensemble for each of the three systems, resulting in a total of ∼1.5 μs of dynamics. Coordinates of the systems were collected every 5 ps for each run. Statistics were collected considering the equilibrated trajectories only, thus discarding the first ∼50 ns of simulation for all the systems.

##### Confidence Interval (C.I.) Analysis

To determine whether the *d-newbond* distribution are significantly different with respect to the different mutant systems (human DNA Pol-η K231A, K224A, and K224A/K231A) we performed a confidence interval (C.I.) analysis. The null hyphothesis is expressed by μ_X_=μ_Y_, where μ represents the mean of the distribution, while X and Y are two different systems among the mutated. In order to test the null hypothesis, we computed the distributions of the *d-newbond* differences (d_X-Y_) for all possible cases and evaluated the mean μ and the associated standard error σ_*E*_. Values are reported in in the Table S4, together with the C.I. at which the null hypothesis can be rejected. The populations of *d-newbond* in DNA Pol-η K231A, K224A, and K224A/K231A were 90001, 90001, 83540, respectively, and were obtained by sampling the MD trajectory every 10 ps, for a total of 1.5 μs.

## Author Contributions

M.M. and M.D.V. conceived and supervised the project. M.M. performed sequence and structural alignments. V.G. performed the MD analyses and calculated the electrostatic potential maps. M.C. performed the analyses of K1 and K2 mutants. M.M. and M.D.V. wrote the manuscript with contributions from all authors.

## Supporting Citations

The following references appear in the Supplemental Information: Strathern et al., 2013, Treich et al., 1992.

## Supplemental Information

Document S1. Figures S1–S3 and Tables S2–S4

Table S1. DNA/RNA Processing Enzymes Adopting a Group II Intron-like Architecture of the Active Site, Related to Table 1

Data S1. Sequence Alignments and Conservation of K1-like (in Blue) and K2-like (in Red) Residues for Different Classes of Two-Metal-Ion Enzymes, Related to Table 1

Document S2. Article plus Supplemental Information
