# Supplementary material for: Second-Shell Basic Residues Expand the Two-Metal-Ion Architecture of DNA and RNA Processing Enzymes
Source: Structure. 2018 Jan 2;26(1):40–50.e2. doi: 10.1016/j.str.2017.11.008 (PMC5758106; doi:10.1016/j.str.2017.11.008)
Supplement: Document S1. Figures S1–S3 and Tables S2–S4 [file mmc1.pdf]

Structure, Volume *26*

Supplemental Information

Second-Shell Basic Residues Expand the Two-Metal-Ion Architecture of DNA and RNA Processing Enzymes

Vito Genna, Matteo Colombo, Marco De Vivo, and Marco Marcia

SUPPLEMENTAL INFORMATION FOR:

Second-shell basic residues expand the two-metal-ion architecture of DNA and RNA processing enzymes.

Vito Genna[1,#], Matteo Colombo[2,#], Marco De Vivo[1,3,*], Marco Marcia[2,*]

[*]To whom correspondence should be addressed. E-mail: marco.devivo@iit.it; mmarcia@embl.fr

The supplemental information file contains:

- Fig. S1 (Related to Fig. 1)
- Fig. S2 (Related to Fig. 2)
- Fig. S3 (Related to Fig. 3)
- Table S2 (Related to Table 1)
- Table S3 (Related to Fig. 3)
- Table S4 (Related to Fig. 3)

Supplemental Figures and Tables

Fig. S1: Additional examples of nucleases and polymerases possessing K1- and K2-like residues (Related to Fig. 1). A) Restriction endonuclease EcoRI (PDB id.: 1QPS); B) Restriction endonuclease PvuII (1F0O); C) Endonuclease MutH (2AOR); D) RNase-H (3O3H); E) DNA polymerase Pol-ι (2ALZ); and F) Norwalk virus RdRp (PDB id.: 3BSO). Structural elements are oriented and color coded as in Fig. 1.

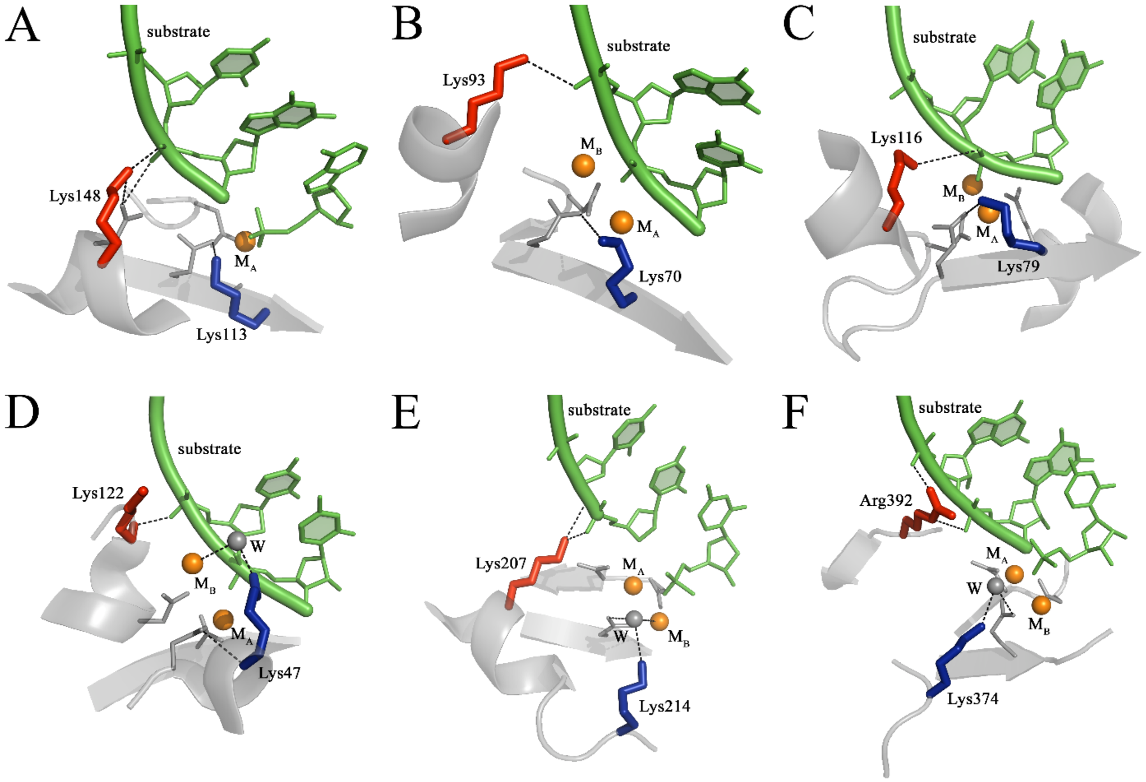

Fig. S2: Structural overlap of K1- and K2-like residues in selected subsets of two-metal-ion enzymes (Related to Fig. 2). **Subset of ribozymes:** overlap of the structures of the group II intron (4FAR) and of the spliceosome (5LJ3, putative K1 ion and K2-like Lys611, see also Fig. 5 for details). Only the exon (substrate) and M_A-M_B center of 4FAR are represented for clarity. **Subset of restriction endonucleases:** overlap of the structures of BamHI (2BAM), PvuII (1F0O), BpuSI (3S1S), MspI (1SA3), FokI (1FOK), and NaeI (1IAW). Only the substrate and M_A-M_B center of 2BAM are represented for clarity. **Subset of Cas9-like nucleases:** overlap of the structures of the RuvC domain of Cas9 nucleases from *S. pyogenes* (4CMQ/5F9R, see also Figure 1C), *A. naeslundii* (4OGC), *F. tularensis* (5B2O), and *S. aureus* (5AXW), and of RNase-H from *T. maritima* (3O3H) and *H. sapiens* (3PUF). Only the substrate and M_A-M_B center of *S. pyogenes* Cas9 are represented for clarity. **Curated subset of evolutionarily-related DNA polymerases:** overlap of the structures of human (PDB id 4ECS) and yeast (3MFI) DNA polymerase η, human DNA polymerase ι (2ALZ), human DNA polymerase κ (2OH2), *S. solfataricus* Dpo4 (2AGO), and *E. coli* DinB (4IRK). Only the substrate and M_A-M_B center of 4ECS are represented for clarity. **Subset of viral RdRps:** overlap of the structures of the RdRps from Norwalk virus (3BSO), Sapporo virus (2UUW), Rabbit haemorragic disease virus (1KHV), Poliovirus (1RDR), Foot-and-mouth disease virus (2E9T), Hepatitis C virus (4E78), and Dengue virus (2J7U). Only the substrate and M_A-M_B center of 3BSO are represented for clarity. **Subset of eukaryotic DdRps:** overlap of the structures of yeast Pol-I (5M5Y), Pol-II (2E2H), and Pol-III (5FJ8). Only the substrate and M_A-M_B center of 2E2H are represented for clarity. The structures were aligned and represented as described in Fig. 2. Sites 3 and 4 are defined in Fig. 4 and in the main text.

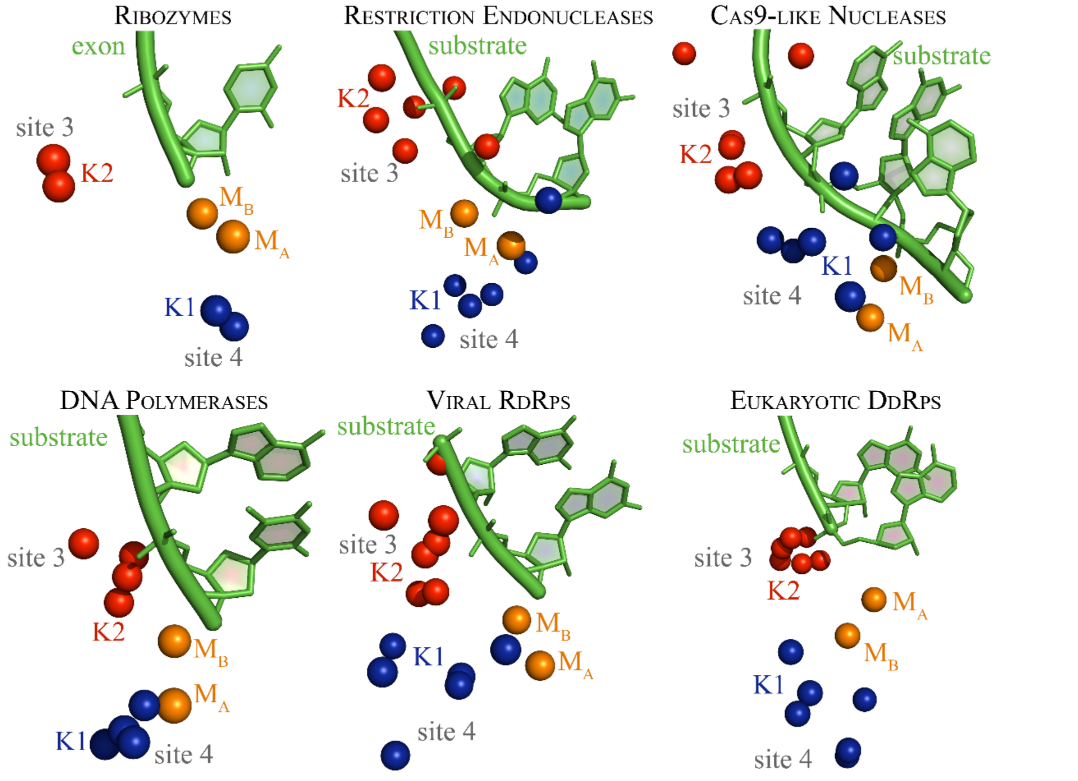

Fig. S3: Structural superimposition of the wild type and the mutated Pol-η in complex with dsDNA and dATP from molecular dynamics simulations (Related to Fig. 3). A) Superimposition between human wild-type DNA Pol-η (white) and K231A mutant (grey). B) Superimposition between human wild-type DNA Pol-η (white) and K224A mutant (ochre). C) Superimposition between human wild-type DNA Pol-η (white) and K224A/K231A mutant (cyan). Spheres indicate magnesium ions while the nucleic acid substrate, dATP, and the K1- and K2-like residues are reported in licorice. These superpositions show the significant distortion of the reactant state upon mutations of K1- and/or K2-like residues.

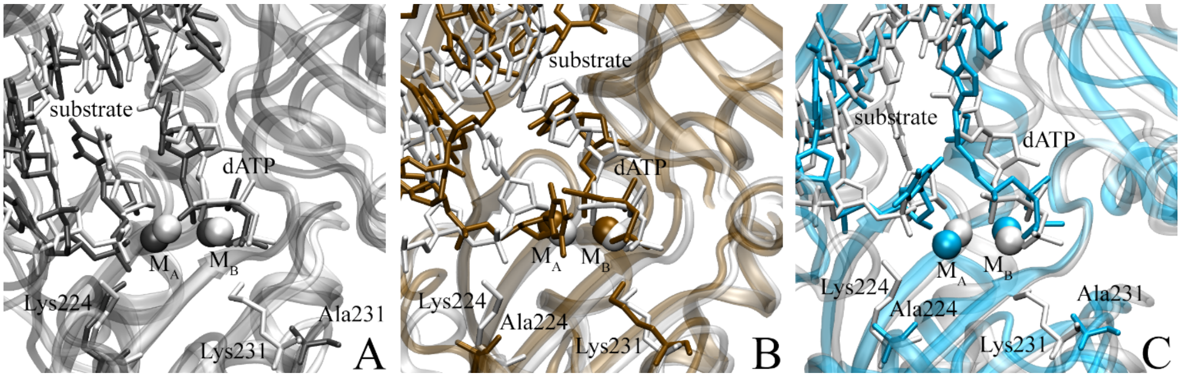

Table S2. Prediction of K1- and K2-like residues in other classes of two-metal-ion enzymes (Related to Table 1).

Enzyme	PDB id	Resolution (Å)	Enzymatic Classification (E.C.)	K1	d1 (Å)[a]	d-ac (Å)[b]	mutants	functional defect	K2	d2 (Å)[a]	d-sub (Å)[c]	mutants	functional defect
Spliceosome (*S. cerevisiae*)	5LJ3	3.80	ribozyme	K1?[d]	~3.8	~2.6	n.a.	n.a.	Lys611 (Prp8)	9.08	3.67	n.a.	n.a.
									Arg614 (Prp8)	9.88	3.38	n.a.	n.a.
RNA Pol-I (*S. cerevisiae*)	5M5Y	4.00	2.7.7.6	Lys934 (RPA190)	x[e]	8.49	n.a.	n.a.	Lys916 (RPA135)	x[e]	3.44	n.a.	n.a.
				Arg957 (RPA135)	x[e]	5.13	n.a.	n.a.	Lys924 (RPA135)	x[e]	2.36	n.a.	n.a.
RNA Pol-II (*S. cerevisiae*)	2E2H	3.95	2.7.7.6	Lys752 (Rbp1)	5.40	4.03	K752L (Strathern et al., 2013)	Lethal	Lys979 (Rbp2)	8.86	2.58	K979R (Treich et al., 1992)	Lethal
				Arg1020 (Rbp2)	3.12	3.57	n.a.	n.a.	Lys987 (Rbp2)	7.13	3.34	K987R (Treich et al., 1992)	Lethal
RNA Pol-III (*S. cerevisiae*)	5FJ8	3.90	2.7.7.6	Lys800 (Rpc1)	x[e]	9.71	n.a.	n.a.	Lys911 (Rpc2)	x[e]	3.75	n.a.	n.a.
				Arg952(Rpc2)	x[e]	7.55	n.a.	n.a.	Lys919 (Rpc2)	x[e]	4.45	n.a.	n.a.

[a] closest distance between the ion / amino acid corresponding to K1 or K2 and the M_A-M_B center; [b] closest distance between the ion / amino acid corresponding to K1 and the acidic residues that chelate M_A-M_B; [c] closest distance between the ion / amino acid corresponding to K2 and the substrate; [d] putative, see main text for details; [e] no substrate or ions in the active site; n.a. = not available;.

Table S3: Calculated RESP charges (Related to Fig. 3). Left: The QM electrostatic potential (ESP), required during the RESP fitting procedure to derive dATP atom charges, was calculated at the HF/6-31G* level of theory by using Gaussian 09. Right: Corrected charges for metal ions and ligands with respect to AMBER RESP charges. Inset: ball and stick representation of dATP with corresponding atomic nomenclature.

Label	Charge
PA	1.630320
PB	1.752583
PG	1.631219
C5’	0.145097
O5	-0.653778
C4’	0.080100
O4	-0.436726
C3’	0.079202
O3	-0.651155
C2’	0.114262
C1’	0.217376
N1	-0.864491
O1A	-0.885583
O1B	-0.880119
O1G	-0.897715
C2	0.622012
O2A	-0.895529
O2B	-0.855092
O2G	-0.846348
N3	-0.797823
O3A	-0.899244
O3B	-0.903616
O3G	-0.883397
C4	0.430437
C5	-0.267981
C6	0.685211
N6	-1.042088
N7	-0.584816
C8	0.346741
N9	-0.312571
H01	0.060952
H02	0.068952
H03	0.083337
H04	0.058165
H05	0.387645
H06	0.063558
H07	0.063558
H08	0.135477
H22	0.400770
H13	0.051333
H25	0.043781
H27	0.406164

Atom	δq	q^{eff}	q^{*}_{AMBER}
Mg_A-Mg_B	-0.25	1.75	2.00
Oδ1-2 (Asp13)	0.05	-0.74	-0.80
Oδ1-2 (Asp115)	0.07	-0.73	-0.80
Oε1-2 (Glu116)	0.05	-0.78	-0.82
O (Met14)	0.06	-0.50	-0.56

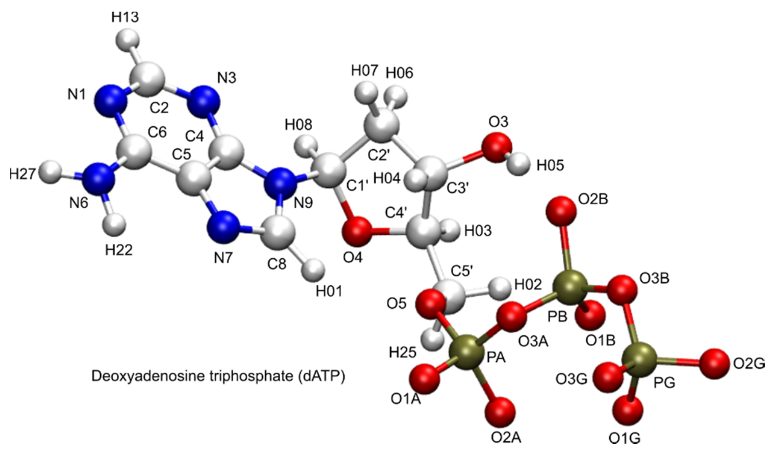

Table S4: Calculated d-*newbond* differences (Related to Fig. 3). Distributions of d-*newbond* differences ($d_{X\text{-}Y}$) for all MD systems with mean μ, standard error σ_E, and confidence interval (C.I.).

	**μ (Å)**	**σ_E (Å)**	**C.I.**
d-*newbond* (K2-K1)	0.02	0.0000447	> 99%
d-*newbond* (K1K2-K2)	0.14	0.0000545	> 99%
d-*newbond* (K2-K1K2)	0.14	0.0000545	> 99%
