# Supplementary material for: Second-Shell Basic Residues Expand the Two-Metal-Ion Architecture of DNA and RNA Processing Enzymes
Source: Structure. 2018 Jan 2;26(1):40–50.e2. doi: 10.1016/j.str.2017.11.008 (PMC5758106; doi:10.1016/j.str.2017.11.008)
Supplement: Data S1. Sequence Alignments and Conservation of K1-like (in Blue) and K2-like (in Red) Residues for Different Classes of Two-Metal-Ion Enzymes, Related to Table 1 [file mmc3.zip › K1K2_Structure_R3_DataFile1.docx]

**Newly-identified structural elements expand the two-metal-ion architecture of DNA and RNA processing enzymes.**

Vito Genna^1,#^, Matteo Colombo^2,#^, Marco De Vivo^1,3,*^, Marco Marcia^2,*^

^*^To whom correspondence should be addressed. E-mail: marco.devivo@iit.it; mmarcia@embl.fr

**Data File 1: sequence alignments and conservation of K1-like (in blue) and K2-like (in red) residues for different classes of two-metal-ion enzymes** (Related to Table 1).

1. Multiple sequence alignment of restriction endonuclease BamHI

Legend

>B_amyloliquefaciens >2BAM:B|PDBID|CHAIN|SEQUENCE

>B_subtilis >gi|503118864|ref|WP_013353547.1|

>B_cereus >gi|822525455|ref|WP_046955369.1|

>G_stearothermophilus >gi|696476303|ref|WP_033015965.1|

>O_valericigenes >gi|503884873|ref|WP_014118867.1|

>Scytonema_sp_HK-05 >gi|1121323857|ref|WP_073633968.1|

>M_vaginatus >gi|493682480|ref|WP_006632638.1|

>N_piscinale >gi|1011379467|ref|WP_062292232.1|

>T_bouteillei >gi|740242599|ref|WP_038083624.1|

>Marinomonas_sp_S3726 >gi|800993396|ref|WP_046019508.1|

>Nostoc_sp_NIES-3756 >gi|1056316658|ref|WP_067770513.1|

Alignment

B_amyloliquefaciens MEVEKEFITDEAKELLSKDKLIQQAYNEVKTSICSPIWPATSKTFTINNTEKNCNGVVPI 60

B_subtilis MEVEKEFITDEAKELLSKDKLIQQAYNEVKTSICSPIWPATSKTFTINNTEKNCNGVVPI 60

B_cereus MKIEKEFITETAKNLLLTDSLIEQAYKEVKTSICSPVWPMESKIFTVNNTKKNCNGVVPI 60

G_stearothermophilus MKVDKVYMTDIAKQLITSDKLCKQAYEEVITSIRSSVWPKGSNIFTINNSEKNVNGVVPL 60

O_valericigenes MKIAKFYISPVADNLMRTCPAAVEAYHEVEQSIIENTTP-GKDIFILNDTSKNCNGVVPV 59

Scytonema_sp_HK-05 MKIVQE-VSLISIGSFEESSDWSIIRSEIRSAISLIVYPPGTSSFTINPTKHG-NGVKPI 58

M_vaginatus MKIVQE-VSLISRGSFEESEEWGVIKNEIRIAIDAIAWPVGASNFTINPTRHG-NGVKPI 58

N_piscinale MKIVQE-VSLISIGSFEESSDWAIIRSEIRDAIALIVHPTGTSSFTINPKKHG-NGVKPI 58

T_bouteillei MKIVQE-VSLMSRGSFEKSQQWVTIQNEIRSAIQLIVWPPGTSNFTINPTPHG-NGVKPI 58

Marinomonas_sp_S3726 MKITRT-EVLINSGGFFDTQQFNDVLAEIEESISKVVWPLNSQYFSINPTKKG-NGVKPI 58

Nostoc_sp_NIES-3756 MKIVQE-VSLINIGSFAESSDWSIIRAEIRNAISVIVHPPGTSSFTINPKKHG-NGVKPI 58

*:: : : *: :* * . * :* . :. *** *:

B_amyloliquefaciens KELCYTLLEDTYNWYREKPLDILKLEKK-KGGPIDVYKEFIEN----------------S 103

B_subtilis KELCYTLLEDTYNWYREKPLDILKLEKK-KGGPIDVYKEF---------IENSE------ 104

B_cereus KELCYTTLEETYNWYREKPLNVLKVEKK-KGGPIDVYKEFSAGLSEKKELDNLEGLNQVD 119

G_stearothermophilus KENCYIMLEETYNWFREKPLDVLKYEKK-KGGPIDVYKEF---------RDGDT------ 104

O_valericigenes KERCYQILEEDHLWYREKPLSYFHDDAQ-KGGPIDVYKEF---------RTPSG------ 103

Scytonema_sp_HK-05 KEACMTTLRDQFGWRLETPI---RYATK-SPGKVDATK-V---------IDDYL------ 98

M_vaginatus KNACMAALHDNFGWQLETKI---RFATR-APGRVDATK-I---------LDNHL------ 98

N_piscinale KEACMIALRDRFGWRLEAPI---NYATK-SPGKVDATK-V---------IDNYF------ 98

T_bouteillei KNACMAFLKETFGWQLETKI---TYATK-SPGRVDATK-A---------LNGHL------ 98

Marinomonas_sp_S3726 KNGCMSHL-EGLGWQLEERL---RITSTMRPGPLDAVKTL---------PNGSV------ 99

Nostoc_sp_NIES-3756 KEACMLALKDKFDWRLETAI---NYATK-SPGKVDATK-V---------IDNHL------ 98

*: * * : * * : * :*. *

B_amyloliquefaciens ELKRVGMEFETGNISSAHRSMNKLLLGLKHGEIDLAIILMPIKQLAYYLTDRVTNFEELE 163

B_subtilis -LKRVGMEFETGNISSAHRSMNKLLLGLKHGEIDLAIILMPIKQLAYYLTDRVTNFEELE 163

B_cereus NIRRVGMEFETGNISSAHRSMNKLLLGLKREEIDLAIILMPIKKLAYYLTDRVTNFEELE 179

G_stearothermophilus -VRRVGLEFETGNISSAHRSMQKLLLGLNRKELDMAIILMPVFELAYYLTDRVTNYEELE 163

O_valericigenes -FFRAGLEFETGNISSAHRSMNKLCVGILKGEIDLAMLMMPIKQMSYYLTDRVSNYEELE 162

Scytonema_sp_HK-05 ----FALEWETGNISSSHRAVNKLVLGLLRGVFLGAALVLPSRKLYPYLTDRIGNYEELE 154

M_vaginatus ----FAFEWETGNISSSHRAVNKLVLGILRGIFLGTALVLPSRQLYPYLTDRIGNYEELE 154

N_piscinale ----FALEWETGNISSSHRAVNKMVLGLIRGVFLGAALVLPSRKLYPYLTDRIGNYEELE 154

T_bouteillei ----FALEWETGNISSSHRAVNKLVLGLLRGVFLGSALVLPSRKLYPYLTDRVGNYEELE 154

Marinomonas_sp_S3726 ----LALEWETGNISSSHRALNKMVLGILEGALTGGILILPSRAMYKYLTDRIGNFQEIE 155

Nostoc_sp_NIES-3756 ----FALEWETGNISSSHRAVNKLVLGLLRGIFLGAALVLPSRKLYPYLTDRIGNYEELE 154

.:*:*******:**:::*: :*: . : :::* : *****: *::*:*

B_amyloliquefaciens PYFEL------TEGQPFIFIGFNAEAYNSNVPLIPKGSDGMSKRSIKKWKDKVENK 213

B_subtilis PYFEL------TEGQPFIFIGFNAEAYNSNVPLIPKGSDGMSKRSIKKWKDKVENK 213

B_cereus PYFEL------TEGQPFMFIGFDAEAYNVNVPVIPKGSDGMSDRSIKKWKDKIEDL 229

G_stearothermophilus PYFEN------AEGKAFVFIGFNADAFDSSVEIIPKGKDGMSKRSIKKWIQKKD-- 211

O_valericigenes PYFILL------DNYPFVVFGFDAEQYRNQAPFLPKGKDGMSPRTKRKWQNNHD-- 210

Scytonema_sp_HK-05 PYFDVWRAVRIQEGFLAIFV-IEHDQVDTSVPRITKGTDGRALV------------ 197

M_vaginatus PYFDVWRAVNIQEGFLAIFV-IEHDALDSSVPTITKGTDGRALI------------ 197

N_piscinale PYFDVWRSVQLQEGFLAIFV-IEHDQLDSNVPTLTKGTDGRALV------------ 197

T_bouteillei PYFDVWRSVNINEGFLAIFV-VEHDAQDSNVARITKGTDGRALI------------ 197

Marinomonas_sp_S3726 PYFPQWRRPDISNGYLAVIE-IEHEYEDENSPLIPKGTDGWAEFQGS--------- 201

Nostoc_sp_NIES-3756 PYFDVWRSVHLSEGFLAIFV-IEHDQLDSNVPTLSKGTDGRALI------------ 197

*** :. :. .: : . : **.** :

1. Multiple sequence alignment of restriction endonuclease BglI

Legend

>B_subtilis >1DMU:A|PDBID|CHAIN|SEQUENCE

>B_atrophaeus >gi|737721210|ref|WP_035690053.1|

>Y_intermedia >gi|912843510|ref|WP_050288336.1|

>N_bacterium_HCH-1 >gi|983041745|gb|KWT84937.1|

>Geitlerinema_sp_PCC7105 >gi|1102700459|ref|WP_071592032.1|

>N_eutropha >gi|1124648614|ref|WP_074928903.1|

>Planktothricoides_sp_SR001 >gi|935599881|ref|WP_054465660.1|

>P_agardhii >gi|754791914|ref|WP_042155505.1|

>Cyanobacterium_ESFC-1 >gi|517210223|ref|WP_018399041.1|

Alignment

B_subtilis MYNLHREKIFMSYNQNKQYLEDNPEIQEKIELYGLNLLNEVISDNEEEIRADYNEANFLH 60

B_atrophaeus MYNLHREKIFMSYNQNKQYLEDNPEIQEKIELYGLNLLNEVISDNEEEIRADYNEANFLH 60

Y_intermedia MFNINRFQQYEAYNENRAYLIDNPDVLINLERFFLIKISELIKQHSLGIQSDYNEASFLY 60

N_bacterium_HCH-1 MFNKFRKEQQKIYINIRNHLSNRPEILINLENYFADYTGKLLQENIESIKSDYNEASYLY 60

Geitlerinema_sp_PCC7105 MFNTFRELQYYSYNRARNYFRDNYIQLVKLENFLSKQVFQLIQNYIIQIKEDYNEASYLY 60

N_eutropha MINIHRQNQFNIYNSARNHFIANPSSLIELEKFLTNYLVSIITANIVEIKQDYNEASYLY 60

Planktothricoides_sp_SR001 MFNKFRNSQYSIYKRARKYFIQNYNQLIDIEKFVSIKFYEIVNNNLQQIVSDFNEASNLY 60

P_agardhii MFNKFRNSQYSIYNQARNYFIQNYDQLIGIEKFIALKIYEIVNNNIQQIANDFNEASNLY 60

Cyanobacterium_ESFC-1 MFNKFRKSQYSLYNQARTHFLQHYNQLINIEKFVSTKIDEIINKNLQQIVNDFNEASNLY 60

* * * * : :: . :* : .:: * *:***. *:

B_subtilis PFWMNYPPLDRGKMPKGDQIPWIEVGEKAVGSKLTRLVSQREDITVREIGLPTGPDERYL 120

B_atrophaeus PFWMNYPPLDRGKMPKGDQIPWIKVGEKAVGSKLTRLVSQREDITVREIGLPTGPDERYL 120

Y_intermedia PFWQNYPPDNRGRQPRGDQYPWIEVGEHSVGRKLSRHLA--EHFFVKDIGVPTGADERFL 118

N_bacterium_HCH-1 PFWQQYPPDNRGRQPRGDQFPWIEVGEHAIGDKLPRLFQ--KDFSLRDVGLPTGPDKRFI 118

Geitlerinema_sp_PCC7105 PFWQNYPPEERGRQPIGDQYPWIEVGEHSLGGKLYRLLS--LSFNIRDIGLPAGSDIRVV 118

N_eutropha PFWENYPPEDRGRQPIKDQYPWIEVGEHAIGSKLPRLLDS--VFRVRDTGLPTGSDQRFV 118

Planktothricoides_sp_SR001 PFWQNYPPDDRGRSPIGDQYPWIEVGEHTIGYKLPRLLE--PYFRIRDIGLPSGSDLRLV 118

P_agardhii PFWQNYPPEERGRYPIGDQYPWIEVGEHSIGDKLPRLLE--PYFSIRDVGLPTGADVRLV 118

Cyanobacterium_ESFC-1 PFWQNYPPDNRGRAPIGDQYPWIEVGEHTIGEKLPRLLE--PYFHIRDMGLPSGTDVRLV 118

*** :*** :**: * ** ***:***:::* ** * . : ::: *:*:* * * :

B_subtilis LTSPTIYSLTNGFTDSIMMFVDIKSVGPRDSDYDLVLSPNQVSGNGDWAQLEGGIQNNQQ 180

B_atrophaeus LTSPTIYSLTNGFTDSIMMFVDIKSVGPRDSDYDLVLSPNQVSGNGDWAQLEGGIQNNQQ 180

Y_intermedia ISSKEILNYSGGMYENVFLSIDIKSVGPRDDAHHAVMSHNQISGDGCWDNIEKGVYNTPL 178

N_bacterium_HCH-1 VTSPLIAEIT-GFTNSAWLFIDIKSVGPRDDADHTVMSHNQISGDGTWTDLEKGIKNSVM 177

Geitlerinema_sp_PCC7105 LSSDKIYQITEGFTNSCWLFVDIKSVGPRDDWNHAVMSHNQISGSGRWDSLLSGITNDVI 178

N_eutropha LTDDAIATATGGFTNSVWFFVDIKSVGPRDDQHHTVMSHNQVSGDGVWINPVDGVRNTIL 178

Planktothricoides_sp_SR001 LTHSEINKLTNSFTDTCWLFLDIKSVGPRDDQNHAVMSPNQISGSGRWDSADSGVVNDVI 178

P_agardhii LTHPEINNLTNSFTDTCWLFLDIKSVGPRDDQSHAVMSPNQISGSGIWDSVDGGVSNTVI 178

Cyanobacterium_ESFC-1 LTNAEINKLTNRLTDTCWLFLDIKSVGPRDDQNHAVMSPNQISGNGRWDAEDNGVVNDVI 178

:: * : : :. : :*********. . *:* **:**.* * *: *

B_subtilis TIQGPRSSQIFLPTIPPLYILSDGTIAPVVHLFIKPIYAMRSLT---KGDTGQSLYKIKL 237

B_atrophaeus TIQGPRSSQIFLPTIPPLYILSDGTIAPVVHLFIKPIYAMRSLT---KGDTGQSLYKIKL 237

Y_intermedia VARGIRAYHDFHCSLPPLYVLSDRTVAPVITIVVKPVYEMYSLYPTANYQSGQPLKRISL 238

N_bacterium_HCH-1 TAIGSREHHSFYCSIPPLYVLSDRTVAPVVILAIKPVYKMLGL--DNNTVSGQPLSRLEL 235

Geitlerinema_sp_PCC7105 IACGKRSSHPFYCSIPPIYVLSDGTVVPVVIIILKPVYDMLSLE-FDVSDGGQPLSRISC 237

N_eutropha QATGARASHDFHASLPPVFVLSDGTIAPLVMIALKPVYRMLQPNVVGARNDGQPLERIDI 238

Planktothricoides_sp_SR001 VAKGKRKSQAFYCSIPPIYILSDGTMIPVIILIVKPVYRMLSLE-ENSKDGGQPLGRISL 237

P_agardhii VAKGRNKSHLFHASIPPIYILSDGTVIPVIIVILKPVYKMLSLE-EQSEDGGQPLGRISF 237

Cyanobacterium_ESFC-1 VAKGKRKSQDFYCSIPPIYVLSDGTILPVIILIVKPVYRMLSLE-ENSKDGGQPLGRISL 237

* . : * ::**:::*** *: *:: : :**:* * ** * ::.

B_subtilis ASVPNGLGLFCNPGYAFDSAYKFLFRPGKDDRTKSLLQKRVRVDLRVLDKIGP-RVMTID 296

B_atrophaeus ASVPNGLGLFCNPGYAFDSAYKFLFRPGKDDRTKSLLQKRVRVDLRVLDKIGP-RVMTID 296

Y_intermedia ASIPNGLLLNVNPNYL--GLYPGLFYPGKDDKGKSPLKVRARVDFNILQKIASWRYYDIF 296

N_bacterium_HCH-1 VSIPNGLLMEVNPRYL--KKYPNLLYPGKDDKSKNPLKMRCRISFALLREIAAWRVQEFL 293

Geitlerinema_sp_PCC7105 ATVPNGLLLCERPNYL--SEFPELFFPGKDDKTKNPRKKRCRVSFEILKQIEYWRFREIL 295

N_eutropha ACIPNGLLLTQQPNYL--GAYNGLLFPGKDDKSKDPRKLRARVSFELLKNIAPWRVQTIQ 296

Planktothricoides_sp_SR001 ATVPNGLLLQENPNYL--QQYPNLFFPGKDDRSTNYLKKRCRISFDVLKSIDNWRFKEIV 295

P_agardhii ATVPNGLLLHEQPNYL--AQYPNLFFPGKDDKNTNPQKMRCRVSFEVLKSIANWRFQEIV 295

Cyanobacterium_ESFC-1 ASVPNGILLQENPNYL--QQYPNLFFPGKDDQSTNPLKKRCRISFDVLKSIEAWRFKEIV 295

. :***: : .* * : *: *****: .. : * *:.: :* .* * :

B_subtilis MDK- 299

B_atrophaeus MDK- 299

Y_intermedia F--- 297

N_bacterium_HCH-1 ISDA 297

Geitlerinema_sp_PCC7105 VS-- 297

N_eutropha VPFP 300

Planktothricoides_sp_SR001 LP-- 297

P_agardhii LK-- 297

Cyanobacterium_ESFC-1 LP-- 297

.

1. Multiple sequence alignment of restriction endonuclease EcoRI

Legend

>E_coli >1QPS:A|PDBID|CHAIN|SEQUENCE

>S_enterica >gi|970558700|ref|WP_058820205.1|

>A_baumannii >gi|746197619|ref|WP_039255571.1|

>C_achromatium_palustre >gi|860362112|ref|WP_048398711.1|

>R_anatipestifer >gi|491055006|ref|WP_004916653.1|

>P_pleuritidis_F0068 >gi|544002746|gb|ERK02129.1|

>S_suis >gi|758839608|ref|WP_043025063.1|

>S_pneumoniae >gi|895296590|emb|COC92604.1|

>S_moniliformis >gi|1035745704|ref|WP_064580150.1|

Alignment

E_coli ----------------SQGVIGIFGDYAKAHDLAVGEVSKLVKKALSNEYPQLSFRYRDS 44

S_enterica MANKNQSNRLTDQHKLSQGVIGIFGDYAKAHDLAVGEVSKLVKDALGKEYPQLSFRYRDS 60

A_baumannii MANKNQSNRLTDQHKLSQGVIGIFGDYAKAHDLAVGEVSKLVKDALGKEYPQLSFRYRDS 60

C_achromatium_palustre MAKNNQSNRLTSQHKDSHGIVGIFGLKAKYHDMTVEKISHSVIKQLKNEYPQLSFRYRTS 60

R_anatipestifer MAKKNQSTRLTVQHKKSQGVVGIFGEKAKLHDLTLGEISHLVIKQLEEEYPQLTFQYKTS 60

P_pleuritidis_F0068 --------------------------------MTVGEVSHLALKQLQEEYPQLEFQYRTS 28

S_suis MAKKNQSSRLTNQHKASKGVVGIFGDEARTHDSAVGTISHLVKYELEQKYPKLEFRFRKS 60

S_pneumoniae MAKKNQSNRLTNQHKDSRGVVGIFGEDAKSHDIAVGTISHLVKSKLEELYPMLEFRFRKS 60

S_moniliformis M-KKGQSNRLTEQQKEGQGPITIFHEDAQVHDKEVYNTSITVKEKLEEEFPMLTFRYRKD 59

: * . * : :* * *::: .

E_coli IKKTEINEALKKIDPDLGGTLFVSNSSIKPDGGIVEVKDDYGEWRVVLVAEAKHQGKDII 104

S_enterica IRKAEINEALKKIDPELGGTLFVSNSSIKPDGGIVEVKDDNDEWRVVLVTEAKHQGKDII 120

A_baumannii IRKAEINEALKKIDPELGGTLFVSNSSIKPDGGIVEVKDDNDEWRVVLVTEAKHQGKDII 120

C_achromatium_palustre IKKEEINEALKKVDPELGQTLFVSNSSIIPDGGIIEVKDDNSNWRVVLVSEAKHQGKDID 120

R_anatipestifer IRKEEINKALRKIDGELGQTLFVPNSSVKPDGGIIEVKDDNGNWRIILVSEAKHQGKDIE 120

P_pleuritidis_F0068 IKKEEINKALKKIDPGLGKTLFVSNSSIIPDGGIVEVKDDNGEWRIVLVTEAKHQGKDIE 88

S_suis ISKKEINDSLRKIDSELGQTLFNHNANIIPDGGIIEVRDDYGNWRVILVTEAKHQGKDIE 120

S_pneumoniae VSKKEINHYLSKLDKDLGKTLFTQNASIIPDGGIIEVKDDSGSWRVVLVTEAKHQGKDIE 120

S_moniliformis LSKKEINESLQKIDTYLGQTLFVDNAKIKPDGGIIEVKDDQGNWRVVLVSEAKHQGKDIE 119

: * ***. * *:* ** *** *:.: *****:**:** ..**::**:*********

E_coli NIRNGLLVGKRGDQDLMAAGNAIERSHKNISEIANFMLSESHFPYVLFLEGSNFLTENIS 164

S_enterica NIKNGILVGKTGSQDLMAAGNAIERSHKNISEIANFMLYESHFPYILFLEGSNFLTETIS 180

A_baumannii NIKNGILVGKTSSQDLMAAGNAIERSHKNISEIANFMLYESHFPYILFLEGSNFLTETIS 180

C_achromatium_palustre NIKNGVLVGKDNNQDLMAAGNAIERSHKNISEIANLMLSESHFPYVLFLEGSNFLTETIS 180

R_anatipestifer NIRKGRLVGKANNQDLMAAGNAIERSHKNISEIANFMLLESHFPYVLFLEGSNFLTETVS 180

P_pleuritidis_F0068 NIKAGKLVGAKNDQDLMAAGNAIERSHKNISEIANLMLAESHFPYVLFLEGSNFLTETIS 148

S_suis NIRAGKLVGKNNDQDLMAAGNAIERSHKNIAELANFMLSEIHFPYVIFLEGSNFLTETIS 180

S_pneumoniae NIKSGKLVGKNNDQDLMAAGNAIERSHKNIAEIANFMLSEEHFPYIIFLEGSNFLTQTIS 180

S_moniliformis NIMVGKLVGKKGNQDLMVAGNAIERAYKNINEIANFMLSERHFPYILFLEGSNFLTQNVT 179

** * *** ..****.*******::*** *:**:** * ****::*********:.::

E_coli ITRPDGRVVNLEYNSGILNRLDRLTAANYGMPINSNLCINKFVNHKDKSIMLQAASIYTQ 224

S_enterica ITRPDGRVVKLEYNSGMLNRLDRLTAANYGLPINSNLCVNKFVKHKDKTIMLQAASIYTQ 240

A_baumannii ITRPDGRVVKLEYNSGMLNRLDRLTAANYGLPINSNLCVNKFVKHKDKTIMLQAASIYTQ 240

C_achromatium_palustre ITRPDGRVVTLEYNSGMLNRLDRLTSANYGMPINTNLCKNKFIKHKDKTIMLQATSIYTQ 240

R_anatipestifer IERPDGRIVRLEYNSGILNRLDRLTAANYGMPINTNLCVNKFIKHKDKTIMLQATSIYTQ 240

P_pleuritidis_F0068 VKRPDGRVVVLEYNSGMLNRLDRLTAANYGMPINKNLCENKFVKHNEKTIMLQAASIYTQ 208

S_suis VERPDGRVVTLEYNSGILNRLDRLTAANYGLPFNTNLCRNKFVKCENRSIMLQAASMYTT 240

S_pneumoniae VIRPDGRSVILEHDSGILNRLDRLTAANYGMPINTNLCKNKFIKNKDKSIMLQATSIYTT 240

S_moniliformis IARPDGREVTLIYKDGALNRLDRLTAANYGMPLNTNLCENRFVNGNDVNIMLQAASIYTK 239

: ***** * * :..* ********:****:*:*.*** *:*:: :: .*****:*:**

E_coli GDGREWDSKIMFEIMFDISTTSLRVLGRDLFEQLTSK------- 261

S_enterica GNGEKWDAKIMFEIMFDVSTSSLRVLGRDLFNQLT------AK- 277

A_baumannii GNGEKWDAKIMFEIMFDVSTSSLRVLGRDLFNQLT------AK- 277

C_achromatium_palustre GNGEKWDIQKMFDIMLEVSKTSLQVLGSDLFNQIT-----KSK- 278

R_anatipestifer GKGEKWDNKEMFDIMLTISKTSLKVLGSDLFNQIT-----KNK- 278

P_pleuritidis_F0068 GNGEKWKVDKMLEIMLDISRTSLQMLGRDLFSQLT--KNKKSK- 249

S_suis GNGSHWESQAMLEIMLDVAQTSLNILGSDLFNQLTIEEEKNEKE 284

S_pneumoniae GDGSH--------------------------------------- 245

S_moniliformis GSGGHWNDNDMINIMLEVARTSLKILGSDIFDQL-RKTTV---- 278

*.* .

1. Multiple sequence alignment of restriction endonuclease PvuII

Legend

>P_vulgaris >1F0O:B|PDBID|CHAIN|SEQUENCE

>Aeromonas_sp_EERV15 >gi|1057711761|ref|WP_068979542.1|

>A_ferrivorans >gi|917378215|ref|WP_051984927.1|

>P_savastanoi >gi|929544999|ref|WP_054083891.1|

>Bradyrhizobium_sp_ORS375 >gi|918664917|ref|WP_052536119.1|

>Synechococcus_sp_PCC8807 >gi|1047320646|ref|WP_065716609.1|

>A_baumannii >gi|914250353|ref|WP_050560546.1|

>R_leguminosarum >gi|739279779|ref|WP_037142586.1|

>Arthrospira_sp_PCC8005 >gi|495328681|ref|WP_008053422.1|

>V_barjaei >gi|1027659813|ref|WP_063605258.1|

Alignment

P_vulgaris ------------------------------------------------------------ 0

Aeromonas_sp_EERV15 ------------------------------------------------------------ 0

A_ferrivorans ------------------------------------------------------------ 0

P_savastanoi ------------------------------------------------------------ 0

Bradyrhizobium_sp_ORS375 ------------------------------------------------------------ 0

Synechococcus_sp_PCC8807 ------------------------------------------------------------ 0

A_baumannii MIEEV-----FARNLKKIREHMNLSQEQLAEKCDLDRTYIGILERGEKVPTLTTVEKCAN 55

R_leguminosarum ------------------------------------------------------------ 0

Arthrospira_sp_PCC8005 ------------------------------------------------------------ 0

V_barjaei MINKEGLLLQLADNVRLLRQELNWTQEYLAEVSDLSPRQISRVENLENEPSLDVVCAIAN 60

P_vulgaris -----------------MSHPDLNKLLELWPHIQEYQDLALKHGINDIFQDNGGKLLQVL 43

Aeromonas_sp_EERV15 ---------MA-H-------PDIEHLRALWPYIREYQSLATKHGINDIFQDNGGKLLQVL 43

A_ferrivorans ---------MTPH-------DDYARLLEIWPSVQEYQALATKHGIDDIFQDNGGKLLQVL 44

P_savastanoi ---------M-PH-------DDYNILTSVWPAVKEYQALATAHGIDDIFQDNGGKLLQVL 43

Bradyrhizobium_sp_ORS375 ---------MKPSSQED--VDEFKRL---WPSIQAYQDLASKHGIQDIFQDNGGKIVQVL 46

Synechococcus_sp_PCC8807 ---------MNFHP-------DKRILDELFPYIQRYQELASKHGINDIFQDNGGKLLQVL 44

A_baumannii ALGVSVIDLLSNDLYLDSFSSDKETLEAIWPFIRKYQELATKNGINDIFQDNGGKLLQVL 115

R_leguminosarum ---------MKPHE-------DKARMDELMPAIKEFQTLATKHGIGDVFQDNGGKLLQVI 44

Arthrospira_sp_PCC8005 ---------MKSHP-------DKAILDELFPYIQQYQALATKHGINDIFQDNGGKLLQVI 44

V_barjaei AFNLHPSKLYEPIF----FESKIEHLNHIFPSIREMELLAKEEGIKDIFQDNGGKLLQVL 116

. : * :: : ** .** *:*******::**:

P_vulgaris LITGLTVLPGREGNDAVDNAGQEYELKSINIDLTKGFSTHHHMNPVIIAKYRQVPWIFAI 103

Aeromonas_sp_EERV15 LITNLKVLPGREGNDAVDLSGQEYELKSINIDLTKAFSTHHHMNPTIIAKYRKVPWVFAI 103

A_ferrivorans LLMGLKILPGREGNDAVDASGREYELKSVNIELTKGFSTHHHMNPTIIAKYRQVPWAFAI 104

P_savastanoi LLMNLRVLPGREGNDAVDSSGREYELKSVNIELTRGFSTHHHMNPVIIAKYRKVPWVFAM 103

Bradyrhizobium_sp_ORS375 LLLGLTNIAGREGNDAVDSDGREYELKSVNIELTTGVSTHHHMNPTIIAKYRQVDWIFAI 106

Synechococcus_sp_PCC8807 LITGLEVLPGREGNDAKDSDGNEYELKSVNIQLTKSFSTHHHINPRIIEKYRKVDWLFAV 104

A_baumannii LNLDLKVLEGREGNDAVDESGQEYELKSLNIELVKGFSTHHHMNPVIIAKYRQVPWIFAI 175

R_leguminosarum LTLGLTVLPGREGNDAKDDEDREYELKSVNLDLTKGFSTHHHLNPVILKKYRQVDWIFAI 104

Arthrospira_sp_PCC8005 LVTGLQIIPGREGNDAKDADGNEFELKSVNIALTKSFSTHHHINPRIIDKYRQVDWIFAV 104

V_barjaei LVTGLKDLPGREGNDAVDANGYEYELKSLNVNLVRGFSTHHHMNPKIIEKYREVDWVFAV 176

* .* : ******* * . *:****:*: *. ..*****:** *: ***:* * **:

P_vulgaris YRGIAIEAIYRLEPKDLEFYYDKWERKWYSDGHKDINNPKIPVKYVMEHGTKIY------ 157

Aeromonas_sp_EERV15 YSGIEIKSIFLLKPDDLEPYYSAWEKKWHDDGNKDINNPKIPVRYVMEVGKQIF------ 157

A_ferrivorans YRHIALQAVYLLEPSDLEFYFTKWEEKWHADGEKDINNPKIPVVYVMKHGRLIHGTSPEI 164

P_savastanoi YRHIELHAVYILEPSDLEFYFSKWEEKWYADGHKDINNPKIPVAYVMQHGRLVYGNAPVF 163

Bradyrhizobium_sp_ORS375 YRNIELQRVYKLSPKDLEPYFSKWEEKWRSDGGKDINNPKIPIKFVKEVGTLVYETDVSV 166

Synechococcus_sp_PCC8807 YSGINLVSIYQLTPADLEFYYEQWERKWNDSGGKDINNPKISLTYVREHGFLIYEA---- 160

A_baumannii YKNIELQAIYRLEPEDLEEMYVKWEEKWHRDGGKDINNPKIPLKYVMEKGKLMSGTVPII 235

R_leguminosarum YRGIELICVHRLKPWQLEPIFERWEKKWHDEGGKDINNPKIPVAYVLEQGELLDGAVPDI 164

Arthrospira_sp_PCC8005 YRGINLLYIYKLTSSDLEYFYRKWEEKWYSKGGKDINNP--------------------- 143

V_barjaei YKDIELQEIWLLTPEDLEFYYDKWTRQWHDRGGKDINNPKIPLTYVQDNGTLVY------ 230

* * : : * :** : * .:* * ******

P_vulgaris ---------------------------------- 157

Aeromonas_sp_EERV15 ---------------------------------- 157

A_ferrivorans SMRRK----------------------------- 169

P_savastanoi QSRRRVFTPPDAA-------GFGPDDI------- 183

Bradyrhizobium_sp_ORS375 T---EIAEDAKEAQDTVKE--------------- 182

Synechococcus_sp_PCC8807 ---------------------------------- 160

A_baumannii PSKTK--KKKDLGGEHLIEGGFKPDED------- 260

R_leguminosarum KYRPSGIVPKKPPID-----PFMPDDEQPEQKAS 193

Arthrospira_sp_PCC8005 ---------------------------------- 143

V_barjaei -------EKSDDGDLYFAD---ILDFT------- 247

1. Multiple sequence alignment of RNase-H

Legend

>T_maritima >3O3H:A|PDBID|CHAIN|SEQUENCE

>F_pennivorans >gi|1028459368|gb|ANE42301.1|

>P_elfii >gi|655460282|ref|WP_028843263.1|

>P_lettingae >gi|500834657|ref|WP_012002944.1|

>M_infera >gi|973111565|gb|KUK68074.1|

>A_tabaci >gi|737464781|ref|WP_035444766.1|

>B_cereus >gi|488066538|ref|WP_002137935.1|

>E_cecorum >gi|828150775|ref|WP_047242528.1|

>A_suicloacalis >gi|1120435097|ref|WP_073297719.1|

>T_halophilus >gi|1007070803|ref|WP_061840533.1|

Alignment

T_maritima ------------------------------------------------------------ 0

F_pennivorans ---------MNE------------------------------------------------ 3

P_elfii ------------------------------------------------------------ 0

P_lettingae ------------------------------------------------------------ 0

M_infera --------------------------------------------MAFASCAEAYEMVMYN 16

A_tabaci MTQPNKKKSISEIKELLKTITDEKDERLA-LIAQDERLGVQKALVSWK------K---AR 50

B_cereus --M--QKMTIHEAECLLQEIMNEEDERFQ-MLVKDERKGVQKLILKWY------KQKELA 49

E_cecorum --MS---ETIAQIKEKLAQIHDAQDAYVL-QLRKDERAGVQKLIQQFE------NRLAKE 48

A_suicloacalis --M---KMTINEIKERLQYVTDYEDEFLL-ACQADERKGVQLAVKQWQ------KRQVAK 48

T_halophilus --MA--KEAISEIKTHLLATETMTDPYVK-QLQMDERKGVQKLLLQLE------RRLAQK 49

*

T_maritima ---------------GIDELYKKEFGIVAGVDEAGRGCLAGPVVAAAVVLEK--EIEGIN 43

F_pennivorans --EKSKKVNWAEE-------KKHIDYSIIGVDEAGRGPLFGPVVAAAVYFDEGVYIEGIA 54

P_elfii --MRSELFAYDRF-------YKQNFGTVIGVDEAGRGCLAGPVVAAAVILEVQ--LD-VF 48

P_lettingae --MRSELFAYDRF-------YKQNFGTVIGVDEAGRGCLAGPVVAAAVILEVQ--LD-VF 48

M_infera FSMKLSEEEFNKLVRF-DAAYRVDHKIVAGVDEAGRGPLAGPVVAAAVIVLNP--VEGVY 73

A_tabaci QKIKEAEEERDRMLVYEKALWEREFHYVAGIDEVGRGPLAGPVVTASVVLPPDVSLVGIR 110

B_cereus QKEREKFLEMSKY---EDELREKGLTYIAGIDEVGRGPLAGPVVTAAVVLPEDFYIPGLN 106

E_cecorum QAMINKAKTMRQF---ENELLAKGYQAICGIDEVGRGPLAGPVVAAAVILPNDELILGLN 105

A_suicloacalis QKLYDRYQRMNQL---EEN-YATTYSLIAGIDEVGRGPLAGPVVAAAVILDQDKQILGLN 104

T_halophilus ETLKEQFYTMQAF---ERSCYKQGHHLIAGIDEVGRGPLAGPVVAAAVILPEGSEILGLN 106

: *:**.*** * ****:*:* . : :

T_maritima DSKQLSPAKRERLLDEIME-KAAVGIGIASPEEIDLYNIFNATKLAMNRALENLS--VKP 100

F_pennivorans DSKALSEKQREELYNEIFARA-KFGLGLATPEEIDLYNIFHATELAMNRALEILSQFVEI 113

P_elfii DSKQLTAQKREELFLQIMNSA-EVGIGIATPEEIDLYNIFNATKIAMNRALASLN--KKD 105

P_lettingae DSKQLTAQKREELFLQIMNSA-EVGIGIATPEEIDLYNIFNATKIAMNRALASLN--KKD 105

M_infera DSKALSRKIRESLFERIIENS-IVGIGLSSPEEIDLINVLAATRLAMNRALSVLS--ERP 130

A_tabaci DSKKLSVSKREKLYEEIMDAAVSVGIGVVDAAQIDELNILQATKAAMKKSIEQLT--VQP 168

B_cereus DSKKLSEAKRERFYDEIKEHAIAIGVGIISPQVIDEINIYQATKQAMLDAVANLS--CTP 164

E_cecorum DSKQLSEKKRESLYQIIQEKAVAIGIGVVDETTIDAINIYQAARLAMTKAVEQLA--VQP 163

A_suicloacalis DSKKLSLAKRVELFTKIKKEAVAVGIGVVSAEEIDKYNILGATKIAMKKAVSNLN--KQP 162

T_halophilus DSKQLSEKKRLDLDNKIKEQATAIGIGEISAEQIDQVNIYQASKMAMTKAVENLA--VKA 164

*** *: * : * .*:* ** *: *:. ** :: *

T_maritima SFVLVNGKGIELSVPGTCLVKGDQKSKLIGAASIVAKVFRDRLMSEFHRMYPQFSFHKHK 160

F_pennivorans KNVFVDGKNLKLNIPAVCVVKGDSKIYQISAASILAKVTRDKIMEKFHAQYPEYNLIKHK 173

P_elfii AYVLVDGKSLNLSQQGVCIVKGDEKSASIAAASIVAKVLRDRIMVAYDRIYPCYGFSKHK 165

P_lettingae AYVLVDGKSLNLSQQGVCIVKGDEKSASIAAASIVAKVLRDRIMVAHDRIYPCYGFSKHK 165

M_infera DYVIVDGKWLRLDVEGECVVRGDRKSASIASASIIAKVFRDRIMDSLDSLYPEYGYRRHK 190

A_tabaci DYLLVDALELPLPIPQTSIIKGDATSLSIAAASIIAKVTRDRMMTEYDELYPGYGFSKNA 228

B_cereus EYLLIDAMKLPTSIPQTSIIKGDAKSVSISAASIIAKVTRDRMMKELGGKYPAYGFEQHM 224

E_cecorum DYLLIDAMELDLDIQQTSLIKGDARSQSIAAASIIAKVYRDHLMVELDKQYPGYGFGKNA 223

A_suicloacalis ELLLVDAVQLQTPIPQETIIKGDLKSNSIAAASIIAKVTRDEMMVEYGREFPGYGFANNA 222

T_halophilus DHLLIDAMSIDINIPQEKIIKGDARSVSIAAASIIAKVYRDQLMKEYHKIHPHYAFDKNA 224

::::. : :::** *.:***:*** **.:* .* : .:

T_maritima GYATKEHLNEIRKNGVLPIHRLSFEPVLELLTDDLLREFFEKGLISENRFERILN-LLGA 219

F_pennivorans GYPTQEHLELLRKYGPTPFHRLSFEPVINLVSKELLDDWLDRRLITEQR-YRHLLNLLEV 232

P_elfii GYGTVHHLNAIREFGPTVFHRLSFSPVLSNLSVKKVHDLFSENINCE-R-AKVILRKLSS 223

P_lettingae GYGTVHHLNAIREFGPTVFHRLSFSPVLSNLSVKKVHDLFSENINCE-R-AKVILRKLSS 223

M_infera GYCTEMHLNALREFGPTTWHRLTYRPIRELIPKELVSRWSEENEVSNARLFRAGLATLEV 250

A_tabaci GYGTQEHLNGLKNQGPSPIHRYSFSPVTQYKK---------------------------- 260

B_cereus GYGTKQHLEAIEVHGVLEEHRKSFAPIKDMIQK--------------------------- 257

E_cecorum GYGTKEHLEGLEKYGVTPIHRKTFAPIKDMI----------------------------- 254

A_suicloacalis GYGTKEHLEAMKRLGITPIHRRSFSPVKKYI----------------------------- 253

T_halophilus GYGTKAHLTGLKEYGITAIHRKSYAPIKKYL----------------------------- 255

** * ** :. * ** :: *: .

T_maritima RKS------------- 222

F_pennivorans DLFGNTRRSKRKTRVK 248

P_elfii S--------------- 224

P_lettingae S--------------- 224

M_infera KN-------------- 252

A_tabaci ---------------- 260

B_cereus ---------------- 257

E_cecorum ---------------- 254

A_suicloacalis ---------------- 253

T_halophilus ---------------- 255

1. Multiple sequence alignment of MutH

Legend

>H_influenzae >2AOR:B|PDBID|CHAIN|SEQUENCE

>H_haemolyticus >gi|822511222|ref|WP_046942105.1|

>S_pneumoniae >gi|987289758|emb|CVP28239.1|

>P_pneumotropica >gi|980941964|ref|WP_059365670.1|

>A_aphrophilus >gi|491980978|ref|WP_005704304.1|

>P_dagmatis >gi|492155710|ref|WP_005765349.1|

>A_paragallinarum >gi|737717974|ref|WP_035686820.1|

>Mannheimia_sp_MG13 >gi|764739431|ref|WP_044470572.1|

>P_multocida >gi|1124583343|ref|WP_074865438.1|

>M_haemolytica >gi|544865651|ref|WP_021279504.1|

>G_anatis >gi|917516715|ref|WP_052123132.1|

Alignment

H_influenzae ----MIPQTLEQLLSQAQSIAGLTFGELADELHIPVPIDLKRDKGWVGMLLERALGATAG 56

H_haemolyticus ---MI-PQTLEQLLSQAQSIAGLTFGELADELHIPVPPDLKRDKGWVGMLLERALGATAG 56

S_pneumoniae ---MI-PQTLEQLLSQAQSIAGLTFGELADELHIPVPIDLKRDKGWVGMLLERALGATAG 56

P_pneumotropica ---MM-PQTLTQLLQRAQSIAGLTFGELADELTIPVPPNLKRDKGWVGMLLETALGATAG 56

A_aphrophilus ---MI--QTEQQLLARAQSIAGMTFGELAQQLQIPVPPNLKRDKGWVGILIEMALGATAG 55

P_dagmatis ---MI-PRTEQELLQKAQNIAGLRLAELAEELYIPIPSDLKRNKGWVGMLIETALGAAAG 56

A_paragallinarum ---MNSPKTEQDLLALAQSLAGLTFGELAQDLSLPVPPNLKRDKGWVGTLLETALGATAG 57

Mannheimia_sp_MG13 --MITSPQSKQQLLHRARTIAGLSFGELAEELNIIVPPDLKRDKGWVGQLIETALGAKAG 58

P_multocida ---MT-PKTEQELLQRAQAIAGLRFAELAQSLHMLVPPDLKRDKGWVGMLIETALGATAG 56

M_haemolytica MQLSTFSPTEQALLSKAEWLAGFTLGEIAEMLHIPIPADLKRDKGWVGTLIETALGAKAG 60

G_anatis -------------MQRAYQLAGRTFVDIAEQLHIVVPQDLKRDKGWVGNLIECALGASAG 47

: * :** : ::*: * : :* :***:***** *:* **** **

H_influenzae SKAEQDFSHLGVELKTLPINAEGYPLETTFVSLAPLVQNSGVKWENSHVRHKLSCVLWMP 116

H_haemolyticus SKAEQDFSHLGVELKTLPINAEGYPLETTFVSLAPLVQNSGVKWENSHVRHKLSCVLWMP 116

S_pneumoniae SKAEQDFSHLGVELKTLPINAEGYPLETTFVSLAPLVQNSGVKWENSHVRHKLSCVLWMP 116

P_pneumotropica SKAEQDFAHLGVELKTLPINEQGFPLETTFVSLAPLTQNSGVQWENSHVRHKLSCVLWIP 116

A_aphrophilus SKAEQDFEHLGIELKTIPINAQGFPLETTFVSLAPLIQNSGVNWQNSHVRHKLSKVLWIP 115

P_dagmatis SKAERDFAHLGIELKTLPINSKGYPLETTFVSLAPLIQNTGVTWQNSHVKHKLSRVLWIP 116

A_paragallinarum SKAEQDFAHLGIELKTIPIDSQGKPLETTFVSLAPLIHNSGITWQSSHVKHKLSKVLWIP 117

Mannheimia_sp_MG13 SKPEQDFAHLGIELKTIPINSQGYPLETTFVSLAPLIQTAGVNWQNSHLRYKLSQVLWIP 118

P_multocida SKAEQDFAHLGIELKTLPINAQGMPLETTFVSLAPLTQNVGVSWENSHVRHKLSKVLWIL 116

M_haemolytica SKAEQDFAHLGIELKTIPVNQKGLPLETTFVSLAPLTQNNGITWETSHVKHKLSRVLWIP 120

G_anatis SKPEQDFAHLGIELKTVPINRFGEPLETTFVSLAPLIDNNGIVWECSHVRYKLSKVLWIP 107

** *:** ***:****:*:: * ************ .. *: *: **:::*** ***:

H_influenzae IEGSRHIPLRERHIGAPIFWKPTAEQERQLKQDWEELMDLIVLGKLDQITARIGEVMQLR 176

H_haemolyticus IEGSRHIPLRERHIGAPILWKPTAEQERQLKQDWEELMDLIVLGKLDQITARIGEVMQLR 176

S_pneumoniae IEGSRHIPLRERHIGAPIFWKPTTGQERQLKQDWEELMDLIVLGKLEQITARIGEVMQLR 176

P_pneumotropica IEGSRHIALRERHIGTPILWQPSSEQEQQLKQDWEELMEYITMGRLDEITARIGEVMQLR 176

A_aphrophilus IEGERHIPLAERHIGAPILWQPSPHQEARLRQDWEELMDYIVLGKLDQITARLGDVLQLR 175

P_dagmatis VEGERQIPLVDRHIGQGILWQPTAEQEWRLQRDWEELMEYITLGKLDQITARLGEVLQLR 176

A_paragallinarum IEGERHIPLAQRHIGQPILWQPSREQEQRLQQDWEELMEYIVFGRLDEITARLGEVLQLR 177

Mannheimia_sp_MG13 IQGERNIPLASRLIGSPILWQPNAEQEAQLQQDWEELMDYIVLGKVHLITAKIGKVLQLR 178

P_multocida VEGERQIPLSERRVGQPILWQPSAQQELRLKRDWEELMEYISLGKLEQINATLGEVLQLR 176

M_haemolytica VEGERKIPLAERHIGQPILWSPTITQESRLQQDWEELMELIVLGELHKINATLGEVLQLR 180

G_anatis IEGERTIPLAQRRVGQPILWQPSAEEEAQLKQDWEELMDLIVLGEVKKIDARIGEVMQLR 167

::*.* * * .* :* *:*.*. :* :*::******: * :*.:. * * :*.*:***

H_influenzae PKGANSRAVTKGIGKNGEIIDTLPLGFYLRKEFTAQILNAFLETKSL---- 223

H_haemolyticus PKGANSRAVTKGIGKNGEIIDTLPLGFYLRKEFTAQILNAFLETKSL---- 223

S_pneumoniae PKGVNSRAVTKGIGKNGEIIDTLPLGFYLRKEFTAQILNAFLETKSL---- 223

P_pneumotropica PKGANSKAITKGIGKNGEVIDTLPLGFYLRKEFTAGILREFLNDEFFRW-- 225

A_aphrophilus PKGANSKALTKGIGKNGEIIDTLPLGFYLRKAFTHEILQQFIQQTI----- 221

P_dagmatis PKGANSRSLTKGIGKKGEIIDTLPLGFYLRKEFTYEILQNFLTN------- 220

A_paragallinarum PKGANSKALTKGIGRNGEIIDTLPLGFYLRKNFTHEILQQFQQPR------ 222

Mannheimia_sp_MG13 PKGANSRAKTKGIGPQGEVIDTLPLGFYLRKEFTATILQNFLQNR------ 223

P_multocida PKGANSKALTRGIGKHGEMIDTLPLGFYLRKTFTAEILQQFLLGTG----- 222

M_haemolytica PKGRNNRSITSAINAKGEIVQSIPLGFYLRKHFTAEILQNFLHCPL----- 226

G_anatis PKGNNNRALTKAIGQQGQVIDTLPLGFYLRKNFTAKILRNFQNNGYSLSPR 218

*** *.:: * .*. :*::::::******** ** **. *

1. Multiple sequence alignment of Exo-λ

Legend

>Escherichia_virus_lambda >4WUZ:A|PDBID|CHAIN|SEQUENCE

>Enterobacteria_phage_HK630 >gi|428782822|ref|YP_007112573.1|

>Stx2-converting_phage_1717 >gi|209447136|ref|YP_002274221.1|

>Achromobacter_sp_ATCC35328 >gi|928599693|emb|CUK07497.1|

>Cronobacter_phage_ENT47670 >gi|431810511|ref|YP_007237585.1|

>S_pneumoniae >gi|1062527211|ref|WP_069289300.1|

>Vibrio_phage_2E1 >gi|1067529875|gb|AOQ26699.1|

>P_multocida >gi|1096225174|ref|WP_071171091.1|

>H_parasuis >gi|737515692|ref|WP_035494618.1|

>H_influenzae >gi|491918549|ref|WP_005671505.1|

>G_anatis >gi|746077620|ref|WP_039145071.1|

Alignment

Escherichia_virus_lambda GSHMTPDIILQRTGIDVRAVEQGDDAWHKLRLGVITASEVHNVIAKPRS----------- 49

Enterobacteria_phage_HK630 ---MTPDIILQRTGIDVRAVEQGEDAWHKLRLGVITASEVHNVIAKPRS----------- 46

Stx2-converting_phage_1717 ---MTPDIILQRTGIDVRAVEQGDDAWHKLRLGVITASEVHNVIAKPRS----------- 46

Achromobacter_sp_ATCC35328 ---MTPDIILQRTGIDVRAVEQGDDAWHKLRLGVITASEVHNVIAKPRS----------- 46

Cronobacter_phage_ENT47670 ---MTPAIILERTGIDVLTVEQGDEAWQRLRLGVITASDVHNVISKPRS----------- 46

S_pneumoniae ---MTPDIILQRTGIDVRAVEQGDDAWHKLRLGVITASEVHNVIAKPRS----------- 46

Vibrio_phage_2E1 -----MINVNHITGVNTFNIEQGTEEWLRHRAGTITASRAHLVIADDITPPMPDDVEIIP 55

P_multocida ----------MIDNLITLDCEQGTEEWLVARLGIPTATGIKNIVTPSG------------ 38

H_parasuis ---------------MTLNCEQGTEEWLTARLGIPTATGVSNIVTPSG------------ 33

H_influenzae ----------MLDKLITLDCEQGTEEWLAARCGIPTATGISNIVTPTG------------ 38

G_anatis ----------MIDGLITLDCEQGTEEWLVARLGIPTATGIKNIVNNSG------------ 38

. *** : * * * **: ::

Escherichia_virus_lambda -------------------------------GKKWPDMKMSYFHTLLAEVCTGVAPEVN- 77

Enterobacteria_phage_HK630 -------------------------------GKKWPDMKMSYFHTLLAEVCTGVAPEVN- 74

Stx2-converting_phage_1717 -------------------------------GKKWPDMKMSYFHTLLAEVCTGVAPEVN- 74

Achromobacter_sp_ATCC35328 -------------------------------GKKWPDMKMSYFHTLLAEVCTGVAPEVN- 74

Cronobacter_phage_ENT47670 -------------------------------GKKWPDMKMSYFHTLLAEVCTGVAPEVN- 74

S_pneumoniae -------------------------------GKKWPDMKMSYFHTLLAEVCTGVAPEVN- 74

Vibrio_phage_2E1 TEKRGVNDVSYNGESFQGTKANCEKWVRSKLEPVMPDGKKSYMLELIAQIATGNVPESAS 115

P_multocida ---------------------------------QKSGGWISYLAELVAESVEGVTEGFK- 64

H_parasuis ---------------------------------KKSSAWTSYLAELVAESIEGLTEGFK- 59

H_influenzae ---------------------------------KKSGSYLPYLAELIAESIEGLKENYK- 64

G_anatis ---------------------------------KKSSGWFTYLAELVAESIEGGNNIIK- 64

. *: *:*: *

Escherichia_virus_lambda AKALAWGKQYENDARTLFEFTSGVNVTESPIIYRDESMRTACSPDGLCSD-GNGLELKCP 136

Enterobacteria_phage_HK630 AKALAWGKQYENDARTLFEFTSGVNVTESPIIYRDESMRTACSPDGLCSD-GNGLELKCP 133

Stx2-converting_phage_1717 AKALAWGKQYENDARTLFEFTSGVNVTESPIIYRDESMRTACSPDGLCSD-GNGLELKCP 133

Achromobacter_sp_ATCC35328 AKALAWGKQYENDARVLFEFTSGVNVTESPIIYRDESMRTACSPDGLCSD-GNGLELKCP 133

Cronobacter_phage_ENT47670 ARALAWGKQYEDDARALFEFTAGVQVTESPIIYKDETMRTACSPDGLCSD-GRGLELKCP 133

S_pneumoniae AKALAWGKQYENDARTLFEFTSGVNVTESPIIYRDESMRTACSPDGLCSD-GNGLELKCP 133

Vibrio_phage_2E1 FKQAEWGHLNEPLARDAFEAKNFCIVTEAGLIYKDESLRCAISPDGLLMDEKQGLEIKSP 175

P_multocida SQHMERGNELEPLARMAYEFETGHDVTQVGGVYLNEKKELMVSPDGLILSHQKGLEIKCP 124

H_parasuis STDMLRGNLLEEQARMAYEFATGNDVVQVGGVYRNADKDMMVSPDGLIPTLRKGLEIKCP 119

H_influenzae SEDMARGNELEPFARAAYEFETGNAVIQVGGVYLNADKDLMISPDGLIPNLRKGLEIKCP 124

G_anatis TVDMERGNELEPKARMAYEFLTDNTVVQVGGVYLNEQKELMISPDGLIPNLKKGLEIKCP 124

*: * ** :* * : :* : ***** .***:*.*

Escherichia_virus_lambda FTSRDFMKFRLGGFEAIKSAYMAQVQYSMWVTRKNAWYFANYDPRMKR---EGLHYVVIE 193

Enterobacteria_phage_HK630 FTSRDFMKFRLGGFEAIKSAYMAQVQYSMWVTRKNAWYFANYDPRMKR---EGLHYVVIE 190

Stx2-converting_phage_1717 FTSRDFMKFRLGGFEAIKSAYMAQVQYSMWVTRKDAWYFANYDPRMKR---EGLHYVVVE 190

Achromobacter_sp_ATCC35328 FTSRDFMKFRLGGFEAIKSAYMAQVQYSMWVTRKDAWYFANYDPRMKR---EGLHYVVIE 190

Cronobacter_phage_ENT47670 FTSRDFMKFRLGGFEAIKSAYMAQVQFSMWVTGKDAWYFSNYDPRMRR---EGLHHVVVE 190

S_pneumoniae FTSRDFMKFRLGGFEAIKSAYMAQVQYSMWVTRKNAWYFANYDPRMKR---EGLHYVVIE 190

Vibrio_phage_2E1 YTTQVHLDTVLNG--KIKPEYLIQCQFSMWVTGWDKWHFCSYDHRLRGSSQNRLHTVVIE 233

P_multocida KM-KTHIKYILEG--GVPSEYIIQVQVAMWVTGYKSWDFVSYCPEYQK---QTLYLYTAT 178

H_parasuis KM-KTHIKYIIEG--VVPSEYIIQVQVALWVTGYDSWDFVSYCPEYQK---QTLFIHTEN 173

H_influenzae QI-KTHIKYLLQG--GVPQEYLIQVQSALWVTGYETWDFVSYCPEYYK---QPFYLFTAQ 178

G_anatis KM-KTHIKYLLES--GVPSEYLMQVQSALWVTGYETWDFVSYCPDYQK---QPLYLYTAE 178

: .:. : . : *: * * ::*** . * * .* : :. .

Escherichia_virus_lambda RDEKYMASFDEIVPEFIEKMDEALAEIGFVFGEQWR-- 229

Enterobacteria_phage_HK630 RDEKYMASFDEIVPEFIEKMDEALAEIGFVFGEQWR-- 226

Stx2-converting_phage_1717 RDEKYMASFDEMVPEFIEKMDEALAEIGFVFGEQWR-- 226

Achromobacter_sp_ATCC35328 RNEKYMASFDEMVPEFIEKMDEALAEIGFVFGEQWR-- 226

Cronobacter_phage_ENT47670 RDEKYMEDFTEAVPEFIEKMDMALAEIGFTFGEQWR-- 226

S_pneumoniae RDEKY--------------------------------- 195

Vibrio_phage_2E1 RDESIMAKFDKYIPKFIDEMDRQLKKLNFEFNDQWREF 271

P_multocida RDEMLMKAFDEYIPQFLKSLRA------LRDG------ 204

H_parasuis PDPVLMKAFDKYIPQFIETLKA------LKVN------ 199

H_influenzae RDPNLMKSFDRLIPEFIKTLKA------YKSTE----- 205

G_anatis RDPILMKAFDKYIPEFLNALKA------LKGENQWQA- 209

:

1. Structure-based sequence alignment of DNA polymerases

Legend

>H_sapiens_Pol-eta >4ECS:A|PDBID|CHAIN|SEQUENCE

>S_cerevisiae_Pol-eta >3MFI:A|PDBID|CHAIN|SEQUENCE

>H_sapiens_Pol-iota >2ALZ:A|PDBID|CHAIN|SEQUENCE

>H_sapiens_Pol-kappa >2OH2:A|PDBID|CHAIN|SEQUENCE

>S_solfataricus_DPO4 >2AGO:A|PDBID|CHAIN|SEQUENCE

>S_solfataricus_DBH >1IM4:A|PDBID|CHAIN|SEQUENCE

>E_coli_DinB >4IRK:A|PDBID|CHAIN|SEQUENCE

Alignment (extract)

H_sapiens_Pol-eta ----------------------------------------DLQLTVGAVIVEEMRAAIER 209

S_cerevisiae_Pol-eta ----------------------------------------DVILALGSQVCKGIRDSIKD 261

H_sapiens_Pol-iota ----------------------------------------HIRLLVGSQIAAEMREAMYN 165

H_sapiens_Pol-kappa HERSISPLLFEESPSDVQPPGDPFQVNFEEQNNPQILQNSVVFGTSAQEVVKEIRFRIEQ 236

S_solfataricus_DPO4 ------------------------------------------DYREAYNLGLEIKNKILE 134

S_solfataricus_DBH -----------------------------------------GNFENGIELARKIKQEILE 140

E_coli_DinB --------------------------------------------GSATLIAQEIRQTIFN 133

. : :: :

H_sapiens_Pol-eta ETGFQCSAGISHNKVLAKLACGLNKPNRQTLVSHG--SVPQLFSQ--MP-IRKIRSLGGK 264

S_cerevisiae_Pol-eta ILGYTTSCGLSSTKNVCKLASNYKKPDAQTIVKND--CLLDFLDCGKF-EITSFWTLGGV 318

H_sapiens_Pol-iota QLGLTGCAGVASNKLLAKLVSGVFKPNQQTVLLPE--SCQHLIHS--LNHIKEIPGIGYK 221

H_sapiens_Pol-kappa KTTLTASAGIAPNTMLAKVCSDKNKPNGQYQILPNRQAVMDFIKD--L-PIRKVSGIGKV 293

S_solfataricus_DPO4 KEKITVTVGISKNKVFAKIAADMAKPNGIKVIDDE--EVKRLIRE--L-DIADVPGIGNI 189

S_solfataricus_DBH KEKITVTVGVAPNKILAKIIADKSKPNGLGVIRPT--EVQDFLNE--L-DIDEIPGIGSV 195

E_coli_DinB ELQLTASAGVAPVKFLAKIASDMNKPNGQFVITP--AEVPAFLQT--L-PLAKIPGVGKV 188

*:: . ..*: .. **: : :: : : .. :*

H_sapiens_Pol-eta LGASVIEILG---------------------------IEYMGELTQFTESQLQSHFGEKN 297

S_cerevisiae_Pol-eta LGKELID-VLDLPHENSIKHIRETWPDNAGQLKEFLDAKVKQSDYDRSTSNIDPLKTADL 377

H_sapiens_Pol-iota TAKCLEA-LG---------------------------INSVRDLQTFSPKILEKELGISV 253

H_sapiens_Pol-kappa TEKMLKA-LG---------------------------IITCTELYQ-QRALLSLLFSETS 324

S_solfataricus_DPO4 TAEKLKK-LG---------------------------INKLVDTLSIEFDKLKGMIGEAK 221

S_solfataricus_DBH LARRLNE-LG---------------------------IQKLRDILSKNYNELEK------ 221

E_coli_DinB SAAKLEA-MG---------------------------LRTCGDVQACDLVMLLKRF-GKF 219

: : . :

1. Multiple sequence alignment of RNA polymerase Pol-II Rbp1 subunit and structure-based sequence alignment of RPA190, Rbp1, and Rpc1 subunits of RNA polymerases I, II, and III.

Legend

>S_cerevisiae_PolII-Rbp1 >2E2H:A|PDBID|CHAIN|SEQUENCE

>S_arboricola_H6_PolII-Rbp1 >gi|401626472|gb|EJS44418.1|

>C_glabrata_CBS138_PolII-Rbp1 >gi|50289967|ref|XP_447415.1|

>H_valbyensis_NRRL-Y-1626_PolII-Rbp1 >gi|1037356197|gb|OBA26116.1|

>Y_lipolytica_PolII-Rbp1 >XP_501909.2 YALI0C16566p

>A_rubescens_DSM1968_PolII-Rbp1 >ODV60282.1

>O_parapolymorpha_DL-1_PolII-Rbp1 >XP_013932372.1

>R_norvegicus_PolII-Rbp1 >gi|109488292|ref|XP_343923.3|

>H_sapiens_PolII-Rbp1 >gi|4505939|ref|NP_000928.1|

>B_taurus_PolII-Rbp1 >gi|329663165|ref|NP_001193242.1|

>O_garnettii_PolII-Rbp1 >gi|395836506|ref|XP_003791195.1|

>M_mulatta_PolII-Rbp1 >gi|355568192|gb|EHH24473.1|

>M_musculus_PolII-Rbp1 >gi|200794|gb|AAA40071.1|

>S_cerevisiae_PolI-RPA190 >5M5Y:A|PDBID|CHAIN|SEQUENCE

>S_cerevisiae_PolIII-Rpc1 >5FJ8:A|PDBID|CHAIN|SEQUENCE

Multiple sequence alignment (extract)

S_cerevisiae_PolII-Rbp1 TMREITETIAEAKKKVLDVTKEAQANLLTAKHGMTLRESFEDNVVRFLNEARDKAGRLAE 734

S_arboricola_H6_PolII-Rbp1 TMREITETIAEAKKKVLDVTKEAQANLLTAKHGMTLRESFEDNVVRFLNEARDKAGRLAE 734

C_glabrata_CBS138_PolII-Rbp1 TMREISETIAEAKQKVEAVTKEAQANLLTAKHGMTLRESFEDNVVRFLNEARDRAGRLAE 734

H_valbyensis_NRRL-Y-1626_PolII-Rbp1 TMLEITEAIAIAKVKVEEVTKEAQENLLSAKHGMTLRESFEDNVVRFLNEARDKAGRSAE 738

Y_lipolytica_PolII-Rbp1 TMRDVTETIEEAKKKVKEIILEAHANTLTAEAGMTMRESFEHNVSRVLNQARDTAGRSAE 739

A_rubescens_DSM1968_PolII-Rbp1 TMKKITTTIAEAKQKVQDLILDAQANRLELEPGMTLRESFESKVSRVLNQARDDAGHCAQ 732

O_parapolymorpha_DL-1_PolII-Rbp1 TMKTITETIAIAKEKVQEVIMDAQKNLLEAEPGMTVRESFEQKVSKLLNEARDSAGKSAE 694

R_norvegicus_PolII-Rbp1 TYQDIQNTIKKAKQDVIEVIEKAHNNELEPTPGNTLRQTFENQVNRILNDARDKTGSSAQ 757

H_sapiens_PolII-Rbp1 TYQDIQNTIKKAKQDVIEVIEKAHNNELEPTPGNTLRQTFENQVNRILNDARDKTGSSAQ 757

B_taurus_PolII-Rbp1 TYQDIQNTIKKAKQDVIEVIEKAHNNELEPTPGNTLRQTFENQVNRILNDARDKTGSSAQ 757

O_garnettii_PolII-Rbp1 TYQDIQNTIKKAKQDVIEVIEKAHNNELEPTPGNTLRQTFENQVNRILNDARDKTGSSAQ 757

M_mulatta_PolII-Rbp1 TYQDIQNTIKKAKQDVIEVIEKAHNNELEPTPGNTLRQTFENQVNRILNDARDKTGSSAQ 767

M_musculus_PolII-Rbp1 TYQDIQNTIKKAKQDVIEVIEKAHNNELEPTPGNTLRQTFENQVNRILNDARDKTGSSAQ 757

* : :* ** .* : .*: * * * *:*::** :* :.**:*** :* *:

S_cerevisiae_PolII-Rbp1 VNLKDLNNVKQMVMAGSKGSFINIAQMSACVGQQSVEGKRIAFGFVDRTLPHFSKDDYSP 794

S_arboricola_H6_PolII-Rbp1 VNLKDLNNVKQMVMAGSKGSFINIAQMSACVGQQSVEGKRIAFGFVDRTLPHFSKDDYSP 794

C_glabrata_CBS138_PolII-Rbp1 MNLKDLNNVKQMVSAGSKGSFINIAQMSACVGQQSVEGKRIGFGFVDRTLPHFSKDDYSP 794

H_valbyensis_NRRL-Y-1626_PolII-Rbp1 VNLKSLNNVKQMVSSGSKGSFINIAQMSACVGQQSVEGKRIPFGFADRTLPHFSKDDYSP 798

Y_lipolytica_PolII-Rbp1 MSLKDLNNVKQMVVAGSKGSFINISQMSACVGQQMVEGKRVPFGFADRTLPHFCKDDYSP 799

A_rubescens_DSM1968_PolII-Rbp1 MNLKELNNVKQMVVSGSKGSFINISQMSACVGQQIVEGKRIPFGFADRTLPHFTKDDFSP 792

O_parapolymorpha_DL-1_PolII-Rbp1 TSLKDSNNVKQMVTAGSKGSYINISQMSACVGQQIVEGKRINFGFADRSLPHFTKDDYSA 754

R_norvegicus_PolII-Rbp1 KSLSEYNNFKSMVVSGAKGSKINISQVIAVVGQQNVEGKRIPFGFKHRTLPHFIKDDYGP 817

H_sapiens_PolII-Rbp1 KSLSEYNNFKSMVVSGAKGSKINISQVIAVVGQQNVEGKRIPFGFKHRTLPHFIKDDYGP 817

B_taurus_PolII-Rbp1 KSLSEYNNFKSMVVSGAKGSKINISQVIAVVGQQNVEGKRIPFGFKHRTLPHFIKDDYGP 817

O_garnettii_PolII-Rbp1 KSLSEYNNFKSMVVSGAKGSKINISQVIAVVGQQNVEGKRIPFGFKHRTLPHFIKDDYGP 817

M_mulatta_PolII-Rbp1 KSLSEYNNFKSMVVSGAKGSKINISQVIAVVGQQNVEGKRIPFGFKHRTLPHFIKDDYGP 827

M_musculus_PolII-Rbp1 KSLSEYNNFKSMVVSGAKGSKINISQVIAVVGQQNVEGKRIPFGFKHRTLPHFIKDDYGP 817

.*.. **.*.** :*:*** ***:*: * **** *****: *** .*:**** ***:.

S_cerevisiae_PolII-Rbp1 ESKGFVENSYLRGLTPQEFFFHAMGGREGLIDTAVKTAETGYIQRRLVKALEDIMVHYDN 854

S_arboricola_H6_PolII-Rbp1 ESKGFVENSYLRGLTPQEFFFHAMGGREGLIDTAVKTAETGYIQRRLVKALEDIMVHYDN 854

C_glabrata_CBS138_PolII-Rbp1 ESKGFVENSYLRGLTPQEFFFHAMGGREGLIDTAVKTAETGYIQRRLVKALEDIMVHYDG 854

H_valbyensis_NRRL-Y-1626_PolII-Rbp1 ESKGFVENSYLRGLTPQEFFFHAMGGREGLIDTAVKTAETGYIQRRLVKALEDIMVHYDG 858

Y_lipolytica_PolII-Rbp1 ESKGFIENSYLRGLTPQEFFFHAMAGREGLIDTAVKTAETGYIQRRLVKALEDVMVQYDG 859

A_rubescens_DSM1968_PolII-Rbp1 ESKGFVENSYLRGLTPQEFFFHAMAGREGLIDTAVKTAETGYIQRRLVKALEDIMVHYDG 852

O_parapolymorpha_DL-1_PolII-Rbp1 ESKGFVENSYLRGLTPQEFFFHAMAGREGLIDTAVKTAETGYIQRRLLKALEDIMVHYDG 814

R_norvegicus_PolII-Rbp1 ESRGFVENSYLAGLTPTEFFFHAMGGREGLIDTAVKTAETGYIQRRLIKSMESVMVKYDA 877

H_sapiens_PolII-Rbp1 ESRGFVENSYLAGLTPTEFFFHAMGGREGLIDTAVKTAETGYIQRRLIKSMESVMVKYDA 877

B_taurus_PolII-Rbp1 ESRGFVENSYLAGLTPTEFFFHAMGGREGLIDTAVKTAETGYIQRRLIKSMESVMVKYDA 877

O_garnettii_PolII-Rbp1 ESRGFVENSYLAGLTPTEFFFHAMGGREGLIDTAVKTAETGYIQRRLIKSMESVMVKYDA 877

M_mulatta_PolII-Rbp1 ESRGFVENSYLAGLTPTEFFFHAMGGREGLIDTAVKTAETGYIQRRLIKSMESVMVKYDA 887

M_musculus_PolII-Rbp1 ESRGFVENSYLAGLTPTEFFFHAMGGREGLIDTAVKTAETGYIQRRLIKSMESVMVKYDA 877

**:**:***** **** *******.**********************:*::*.:**:**

Structure-based alignment (extract)

S_cerevisiae_PolII-Rbp1 AEAKKKVLDVTKEAQANL-------LTAKHGMTLRESFEDNVVRFLNEAR 726

S_cerevisiae_PolI-RPA190 REAAAEVTNLDKDTPADDPELLKRLQEILRDNNKSGILDAVTSSKVNAIT 904

S_cerevisiae_PolIII-Rpc1 EIAYHKCDELITLFNKGE-------LETQPGCNEEQTLEAKIGGLLSKVR 774

* : :: . . . . :: :.

S_cerevisiae_PolII-Rbp1 DKAGRLAEV----NLKDLNNVKQMVMAGSKGSFINIAQMSACVGQQSVEG 772

S_cerevisiae_PolI-RPA190 SQVVSKCVPDGTMKKFPCNSMQAMALSGAKGSNVNVSQIMCLLGQQALEG 954

S_cerevisiae_PolIII-Rpc1 EEVGDVCIN----ELDNWNAPLIMATCGSKGSTLNVSQMVAVVGQQIISG 820

.:. . : * *. .*:*** :*::*: . :*** :.*

S_cerevisiae_PolII-Rbp1 KRIAFGFVDRTLPHFSKDDYSPESKGFVENSYLRGLTPQEFFFHAMGGRE 822

S_cerevisiae_PolI-RPA190 RRVPVMVSGKTLPSFKPYETDAMAGGYVKGRFYSGIKPQEYYFHCMAGRE 1004

S_cerevisiae_PolIII-Rpc1 NRVPDGFQDRSLPHFPKNSKTPQSKGFVRNSFFSGLSPPEFLFHAISGRE 870

.*:. . .::** * . . : *:*.. : *:.* *: **.:.***

1. Multiple sequence alignment of RNA polymerase Pol-II Rbp2 subunit and structure-based sequence alignment of RPA135, Rbp2, and Rpc2 subunits of RNA polymerases I, II, and III

Legend

>S_cerevisiae_PolII-Rbp2 >2E2H:B|PDBID|CHAIN|SEQUENCE

>S_arboricola_H6_PolII-Rbp2 >gi|401623583|gb|EJS41677.1|

>C_glabrata_CBS138_PolII-Rbp2 >gi|50293059|ref|XP_448959.1|

>H_valbyensis_NRRL-Y-1626_PolII-Rbp2 >gi|1037356434|gb|OBA26349.1|

>Y_lipolytica_PolII-Rbp2 >gi|50549809|ref|XP_502376.1|

>A_rubescens_DSM1968_PolII-Rbp2 >gi|1064972083|gb|ODV62510.1|

>O_parapolymorpha_DL-1_PolII-Rbp2 >gi|927376581|ref|XP_013934141.1|

>R_norvegicus_PolII-Rbp2 >gi|300798312|ref|NP_001178807.1|

>H_sapiens_PolII-Rbp2 >gi|4505941|ref|NP_000929.1|

>B_taurus_PolII-Rbp2 >gi|154707850|ref|NP_001092552.1|

>O_garnettii_PolII-Rbp2 >gi|395819924|ref|XP_003783328.1|

>M_mulatta_PolII-Rbp2 >gi|355687402|gb|EHH25986.1|

>M_musculus_PolII-Rbp2 >gi|226958589|ref|NP_722493.2|

>S_cerevisiae_PolI-RPA135 >5M5Y:B|PDBID|CHAIN|SEQUENCE

>S_cerevisiae_PolIII-Rpc2 >5FJ8:B|PDBID|CHAIN|SEQUENCE

Multiple sequence alignment (extract)

S_cerevisiae_PolII-Rbp2 GKTTPISPDEEELGQRT-AYHSKRDASTPLRSTENGIVDQVLVTTNQDGLKFVKVRVRTT 971

S_arboricola_H6_PolII-Rbp2 GKTTPISPDEEELGQRT-AYHSKRDASTPLRSTENGIVDQVLVTTNQDGLKFVKVRVRTT 971

C_glabrata_CBS138_PolII-Rbp2 GKTTPIAPDEEELGQRT-AYHSKRDASTPLRSTENGIVDQVLITTNQDGLKFVKVRVRTT 970

H_valbyensis_NRRL-Y-1626_PolII-Rbp2 GKTTPIKPDNEELGLRT-AFHTKRDASTPLRSTESGIIDQVLITTNEEGLRFVKVRVRTT 1016

Y_lipolytica_PolII-Rbp2 GKTAPIPPDAEELGQRT-KYHTKRDASTPLRSTENGIVDQVLLTTNQEGLRFVKVRMRTT 973

A_rubescens_DSM1968_PolII-Rbp2 GKTTPISPDNEELGRKT-QFHTKRDASMPLRSTENGIVDQVVLTTNHEGLKFVKVRMRTT 966

O_parapolymorpha_DL-1_PolII-Rbp2 GKTVPIPPDTEELGQRT-KYHTKRDASTPLRTTESGIVDQVLLTTNAEGLKFAKVRMRTT 1020

R_norvegicus_PolII-Rbp2 NKSMPTVTQIPLEGSNVPQQPQYKDVPITYKGATDSYIEKVMISSNAEDAFLIKMLLRQT 888

H_sapiens_PolII-Rbp2 GKTVTLPENEDELESTN-RRYTKRDCSTFLRTSETGIVDQVMVTLNQEGYKFCKIRVRSV 926

B_taurus_PolII-Rbp2 GKTVTLPENEDELEGTN-RRYTKRDCSTFLRTSETGIVDQVMVTLNQEGYKFCKIRVRSV 926

O_garnettii_PolII-Rbp2 NKSMPTVTQIPLEGSNVPQQPQYKDVPITYKGATDSYIEKVMISSNAEDAFLIKMLLRQT 888

M_mulatta_PolII-Rbp2 GKTVTLPENEDELESTN-RRYTKRDCSTFLRTSETGIVDQVMVTLNQEGYKFCKIRVRSV 926

M_musculus_PolII-Rbp2 GKTVTLPENEDELESTN-RRYTKRDCSTFLRTSETGIVDQVMVTLNQEGYKFCKIRVRSV 926

.*: : :* : : . :::*::: * :. : *: :* .

S_cerevisiae_PolII-Rbp2 KIPQIGDKFASRHGQKGTIGITYRREDMPFTAEGIVPDLIINPHAIPSRMTVAHLIECLL 1031

S_arboricola_H6_PolII-Rbp2 KVPQIGDKFASRHGQKGTIGITYRREDMPFTAEGIVPDLIINPHAIPSRMTVAHLIECLL 1031

C_glabrata_CBS138_PolII-Rbp2 KVPQIGDKFASRHGQKGTIGITYRREDMPFTAEGIVPDLIINPHAIPSRMTVAHLIECLL 1030

H_valbyensis_NRRL-Y-1626_PolII-Rbp2 KVPQIGDKFASRHGQKGTIGITYRREDMPFTAEGIVPDLIINPHAIPSRMTVAHLIECLL 1076

Y_lipolytica_PolII-Rbp2 KIPQIGDKFASRHGQKGTIGVTYRHEDMPFSAEGVVPDIIINPHAIPSRMTVAHLIECLL 1033

A_rubescens_DSM1968_PolII-Rbp2 KVPQIGDKFASRHGQKGTIGITYRHEDMPFSREGIVPDLIINPHAIPSRMTVAHLIECLL 1026

O_parapolymorpha_DL-1_PolII-Rbp2 KVPQIGDKFASRHGQKGTIGITYRHEDMPFTAQGIVPDLIINPHAIPSRMTVAHLIECLL 1080

R_norvegicus_PolII-Rbp2 RRPEIGDKFSSRHGQKGVCGLIVPQEDMPFCDSGICPDIIMNPHGFPSRMTVGKLIELLA 948

H_sapiens_PolII-Rbp2 RIPQIGDKFASRHGQKGTCGIQYRQEDMPFTCEGITPDIIINPHAIPSRMTIGHLIECLQ 986

B_taurus_PolII-Rbp2 RIPQIGDKFASRHGQKGTCGIQYRQEDMPFTCEGITPDIIINPHAIPSRMTIGHLIECLQ 986

O_garnettii_PolII-Rbp2 RRPEIGDKFSSRHGQKGVCGLIVPQEDMPFCDSGICPDIIMNPHGFPSRMTVGKLIELLA 948

M_mulatta_PolII-Rbp2 RIPQIGDKFASRHGQKGTCGIQYRQEDMPFTCEGITPDIIINPHAIPSRMTIGHLIECLQ 986

M_musculus_PolII-Rbp2 RIPQIGDKFASRHGQKGTCGIQYRQEDMPFTCEGITPDIIINPHAIPSRMTIGHLIECLQ 986

: *:*****:*******. *: :***** .*: **:*:***.:*****:.:*** *

S_cerevisiae_PolII-Rbp2 SKVAALSGNEGDASPFT-DITVEGISKLLREHGYQSRGFEVMYNGHTGKKLMAQIFFGPT 1090

S_arboricola_H6_PolII-Rbp2 SKVAALSGNEGDASPFT-DITVEGISKLLREHGYQSRGFEVMYNGHTGKKLMAQIFFGPT 1090

C_glabrata_CBS138_PolII-Rbp2 SKVAALSGNEGDASPFT-DITVEGISKLLREHGYQSRGFEVMYNGHTGKKLMAQIFFGPT 1089

H_valbyensis_NRRL-Y-1626_PolII-Rbp2 SKVAAINGQEGDASPFV-DVTVDSISDLLRQAGYQSRGFEVMYNGHTGKKLMAQIFFGPT 1135

Y_lipolytica_PolII-Rbp2 SKVSCLSGLEGDATPFT-DVTVDAISKLLRSHGYQSRGFEVMYHGHTGKKIMAQCFLGPT 1092

A_rubescens_DSM1968_PolII-Rbp2 SKVCALMGTEGDATPFNDDITVDAISDILYTFGYQSRGFEVLYNGHTGKKLMAQVFFGPT 1086

O_parapolymorpha_DL-1_PolII-Rbp2 SKVASMRGYEGDATPFT-DLTVDAVSKLLRENGYQSRGFEVMYNGHTGKKLMAQVFFGPT 1139

R_norvegicus_PolII-Rbp2 GKAGVLDGRFHYGTAFG-GSKVKDVCEDLVRHGYNYLGKDYVTSGITGEPLEAYIYFGPV 1007

H_sapiens_PolII-Rbp2 GKVSANKGEIGDATPFNDAVNVQKISNLLSDYGYHLRGNEVLYNGFTGRKITSQIFIGPT 1046

B_taurus_PolII-Rbp2 GKVSANKGEIGDATPFNDAVNVQKISNLLSDYGYHLRGNEVLYNGFTGRKITSQIFIGPT 1046

O_garnettii_PolII-Rbp2 GKAGVLDGRFHYGTAFG-GSKVKDVCEDLVRHGYNYLGKDYVTSGITGEPLEAYIYFGPV 1007

M_mulatta_PolII-Rbp2 GKVSANKGEIGDATPFNDAVNVQKISNLLSDYGYHLRGKEVLYNGFTGRKITSQIFIGPT 1046

M_musculus_PolII-Rbp2 GKVSANKGEIGDATPFNDAVNVQKISNLLSDYGYHLRGNEVLYNGFTGRKITSQIFIGPT 1046

.*. * .: * .*. :.. * **: * : : * **. : : ::**.

Structure-based alignment (extract)

S_cerevisiae_PolII-Rbp2 -PDEEELGQRTAYHSKRDASTPLRSTENGIVDQVLVTTN----QDGLKFV 964

S_cerevisiae_PolI-RPA135 --YFDDTLNKT-------KIKTYHSSEPAYIEEVNLIGDESNKFQELQTV 901

S_cerevisiae_PolIII-Rpc2 SADAPNPNNVNVQTQYREAPVIYRGPEPSHIDQVMMSVS----DNDQALI 896

: : . :..* . :::* : . : :

S_cerevisiae_PolII-Rbp2 KVRVRTTKIPQIGDKFASRHGQKGTIGITYRREDMPFTAEGIVPDLIINP 1014

S_cerevisiae_PolI-RPA135 SIKYRIRRTPQIGDKFSSRHGQKGVCSRKWPTIDMPFSETGIQPDIIINP 951

S_cerevisiae_PolIII-Rpc2 KVLLRQNRRPELGDKFSSRHGQKGVCGIIVKQEDMPFNDQGIVPDIIMNP 946

.: * : *::****:*******. . ****. ** **:*:**

S_cerevisiae_PolII-Rbp2 HAIPSRMTVAHLIECLLSKVAALSGNEGDASPFT----DITVEGISKLLR 1060

S_cerevisiae_PolI-RPA135 HAFPSRMTIGMFVESLAGKAGALHGIAQDSTPWIFNEDDTPADYFGEQLA 1001

S_cerevisiae_PolIII-Rpc2 HGFPSRMTVGKMIELISGKAGVLNGTLEYGTCFG----GSKLEDMSKILV 992

*.:*****:. ::* : .*...* * .: : . : :.: *

S_cerevisiae_PolII-Rbp2 EHGYQSRGFEVMYNGHTGKKLMAQIFFGPTYYQRLRHMVDDKIHARARGP 1110

S_cerevisiae_PolI-RPA135 KAGYNYHGNEPMYSGATGEELRADIYVGVVYYQRLRHMVNDKFQVRSTGP 1051

S_cerevisiae_PolIII-Rpc2 DQGFNYSGKDMLYSGITGECLQAYIFFGPIYYQKLKHMVLDKMHARARGP 1042

. *:: * : :*.* **: * * *:.* ***:*:*** **::.*: **

1. Viral RdRps

Legend

| >Norwalk >3BSO:A\|PDBID\|CHAIN\|SEQUENCE  >Sapporo >2UUW:A\|PDBID\|CHAIN\|SEQUENCE  >RHDV >1KHV:A\|PDBID\|CHAIN\|SEQUENCE  >Polio >1RDR:A\|PDBID\|CHAIN\|SEQUENCE  >FMDV >2E9T:A\|PDBID\|CHAIN\|SEQUENCE  >FluA_PA/Bat >AFC35437.1  >FluB_PA/memphis >AAU94844.1  >FluC_PA/Johannesburg >5D98:D\|PDBID\|CHAIN\|SEQUENCE  >FluA_PB1/Bat >4WSB:B\|PDBID\|CHAIN\|SEQUENCE  >FluB_PB1/memphis >4WRT:B\|PDBID\|CHAIN\|SEQUENCE  >FluC_PB1/Johannesburg >5D98:E\|PDBID\|CHAIN\|SEQUENCE  >FluA_PB2/Bat >4WSB:C\|PDBID\|CHAIN\|SEQUENCE  >FluB_PB2/memphis >4WRT:C\|PDBID\|CHAIN\|SEQUENCE | >FluC_PB2/Johannesburg >5D98:F\|PDBID\|CHAIN\|SEQUENCE  >LACV >NP_671968.1  >Bunyamwera >AKX73301.1  >Hantaan >APH07644.1  >Imjin >AIF28819.1  >RVFV >AOS50855.1  >Uukuniemi >AIU95041.1  >Peanut >NP_619688.1  >Tomato >AMH38315.1  >CCHV >ABW17160.1  >Kupe >ABY82502.1  >Lassa >AHC95546.1  >Junin >ALE15111.1 |
| --- | --- |

Structure-based alignment of RdRps from *Caliciviridae* and *Picornaviridae* (extract)

Norwalk WLLTLCALSEV-TNL----SPDIIQANSLFSFYGDDEIVST-DIK-LDPE 356

Sapporo MIYVAAAILQAYESHNVPYTGNVFQVET-IHTYGGGCMYSVCPATASIFH 362

RHDV WLLWSAAVYKSCAEIGLH-CSNLYEDAP-FYTYGDDGVYAMTPMMVSLLP 369

Polio NLIIRTLLLKTYKGIDL-------DHLK-MIAYGDDVIASY-PHE-VDAS 341

FMDV NIYVLYALRRHYEGVEL-------DTYT-MISYGDDIVVAS-DYD-LDFE 351

: : . : : **.. : :

Norwalk KLTAKLKEYGLKPTRPDKTEGPLVISEDLNGLTFLRRTVTRDP---AGWF 403

Sapporo TVLANLTSYGLKPTAADKSDAIK----PTNTPVFLKRTFTQTP---HGIR 405

RHDV AIIENLRDYGLSPTAADKTEFIDV--CPLNKISFLKRTFELTD---IGWV 414

Polio LLAQSGKDYGLTMTPADKSATFE--TVTWENVTFLKRFFRADEKYPFLIH 389

FMDV ALKPHFKSLGQTITPADKSDKGFVLGHSITDVTFLKRHFHMDYGT--GFY 399

: . * . * .**: **:* .

Norwalk GK-LEQSSILRQMYWTRGP-NHEDPSET-MIPHSQRPIQLMSLLGEAALH 450

Sapporo AL-LDITSITRQFYWLKAN-RTSDPSSPPAFDRQARSAQLENALAYASQH 453

RHDV SK-LDKSSILRQLEWSKTTSRHMVIEETYDLAKEERGVQLEELQVAAAAH 463

Polio PV-MPMKEIHESIRWTKDP-RNT-------------QDHVRSLCLLAWHN 424

FMDV KPVMASKTLEAILSFARRG-T-I-------------QEKLISVAGLAVHS 434

: . : : : : :: . *

Structure-based alignment of premotif A (also known as motif F, PB1 subunit) of RdRps from *Orthomixoviridae*, *Bunyaviridae* and *Arenaviridae* [extract, see also Muller et al., Rift Valley fever virus L segment: correction of the sequence and possible functional role of newly identified regions conserved in RNA-dependent polymerases. J Gen Virol *75 (Pt 6)*, 1345-1352]

FluA_PB1/Bat ALTLNTMTKDAERGKLKRRAIATPGMQIRGFVYFVELLARNICERLEQSGLPVGGNEKKAKLANVIKKMMAKS------ 302

FluB_PB1/memphis ALSLNTMTKDAERGKLKRRAIATAGIQIRGFVLVVENLAKNICENLEQSGLPVGGNEKKAKLSNAVAKMLSNC------ 302

FluC_PB1/Johannesburg ALTINTMAKDGERGKLQRRAIATPGMIVRPFSKIVETVAQKICEKLKESGLPVGGNEKKAKLKTTVTSLNARM------ 295

LACV KFYFTFFNKGQKTSK--DREIFVGEYEAKMCMYAVERIAKERCKLNPDEMISEPGDGKLKVLEQKSEQEIRFLVETTRQ 1018

Bunyamwera DFSFTFFNKGQKTAK--DREIFVGEFEAKMCMYVIERISKERCKLNTDEMISEPGDSKLRILEKKAEEEIRYIVEKTK- 1018

Hantaan KAMARIVRKYQRTEA--DRGFFITTLPTRCRLEIIEDYYDAIAKNISEEYISYGGEKKILAIQGALEKALRWASGESF- 951

Imjin RAQARIVRKHQRTEA--DRGFFITTLPTRVRLEIIEDYYDSISKNVQEEYISYGGEKKILQIQGSLEKALRWASGTSV- 948

RVFV CMHICLFKKQQH-GG--LREIYVMGAEERIVQSVVETIARSIGKFFASDTLCNPPN-KVKIPETHGIRARKQC------ 979

Uukuniemi CMHVCLFKKPQH-GG--LREIYVLGFEERVVQLVIETIARQICKRFKSETLTNPKQ-KLAIPETHGLRAVKTC------ 982

Peanut DFLVSVFEKMQRTKM--DREIYLMSMKTKMMLYFIEHTYKHVAQSDPSEAISISGDYKIKNLASLSYDTITNYN-TALQ 1357

Tomato DFLVSVFEKMQRTKT--DREIYLMSMKVKMMLYFIEHTFKHVAQSDPSEAISISGDNKIRALSTLSLDTITSYN-DILN 1353

Lassa --KFSLSYK-EQVGG--NRELYIGDLRTKMFTRLIEDYFEALSLQLSGSCLNNEKE-----FENAILS---------MK 1182

Junin --KFGLSYK-EQVGS--NRELYVGDLNTKLMTRLVEDFSEAVGSSMRYTCLNSEKE-----FERAICD---------MK 1174

. * :: * : : :* : :

Structure-based alignment of motif D (PB1 subunit) of RdRps from *Orthomixoviridae*, *Bunyaviridae* and *Arenaviridae* (extract)

FluA_PB1/Bat D-IQQ-G--------------------------VNHFYRTCKLVGINMSQKKSYINKTGTFEFTSFFYRYGF-VANFSME 517

FluB_PB1/memphis T-CME-G--------------------------INDFYRTCKLLGINMSKKKSYCNETGMFEFTSMFYRDGF-VSNFAME 516

FluC_PB1/Johannesburg N-IHW-T--------------------------IRRFNAVCKLIGINMSLEKSYGSLPELFEFTSMFFDGEF-VSNLAME 509

LACV --------------MENDK---------IIDFAMKEFERACLTFGCQANMKKTYVTN-CIKEFVSLFNLYGEPFSIYGRF 1255

Bunyamwera --------------LSDEV---------MIQFASDTFETVCLTFGCQANMKKTYITH-TCKEFVSLFNLHGEPLSIYGRF 1232

Hantaan DDGTD-WFLFVSQQIQAGHLHWFSVNTEMWKSMFNLHEHILLLGSIKISPKKTTVSP-TNAEFLSTFFEGCAVSIPFVKI 1188

Imjin GDASD-WFLYVSQKIQAGQLYWCSVNTEMWKTMFNLHEYLLLLGSVKVSPKKTTVSP-TNAEFLSTFFEGCAVSIPFSKI 1187

RVFV EKVLT-RCKVA---------------AA---ICFRMKKELGVYLAIYPSE-KSTANTDFVMEYNSEFYFHTQHVRPTIRW 1205

Uukuniemi KGATG-KYRYL---------------SA---LIFKYKKVIGKYLGIYSSV-KSTNNTLHLLEFNSEFFFHINHNRPLLRW 1210

Peanut KLLSSFNCSSL---------------SE---LLFRSIQSHFKSYCITLNPKKSYASE-SEVEFISERIINGAVIPLYCRH 1571

Tomato KMLTDFSSLSL---------------PE---MLFRSIEAHFKSFCITLNPKKSYASS-SEVEFISERIVNGAIIPLYCRH 1568

Lassa LMQRD-PEEFK---------------TX---LEF--HYYMSNQLNKFISP-KSVIGR-FVAEFKSRFYVWGDEVPLLTKF 1405

Junin EKCQD-RTELL---------------EM---VCF--HEFLSSKLNKFISP-KSVIGT-FVAEFKSRFFVMGEETPLLTKF 1398

. *: *: *

Structure-based alignment of motif G (PA subunit) of RdRps from *Orthomixoviridae*, *Bunyaviridae* and *Arenaviridae* [extract, see also Gerlach et al, (2015) Structural Insights into Bunyavirus Replication and Its Regulation by the vRNA Promoter. Cell *161*, 1267-1279]

FluA_PA/Bat RNRENDWPIGESPQGIEKGTIG----------------K-VCRVLLAKSVFNSI-YASAQLEGFSAESRKLLLLIQAFRD 669

FluB_PA/memphis KGDR-----------VNSPKTFSIGTQEGKLVKGSF-GK-ALRVIFTKCLMHYV-FGNAQLEGFSAESRRLLLLIQALKD 670

FluC_PA/Johannesburg -----------------ELIAQPL-----------R--E-ALRVQLVQQFYFCI-YNDSQLEGFCNEQKKILMALEGDKK 658

LACV --------------------------N-------PT--L-VMSDIMNFSIYTSL-SITKSVLSLTEPARYMIMNSL---- 660

Bunyamwera --------------------------N-------PE--V-SLMDVLNFSFYTSL-SITKSMLSLTEPSRYMIMNSL---- 661

Hantaan -----------T----EDQG-----QF-------PL-QYA-IRSVFANHFLLAI-CQKMKLCAIFDNLRYLIPAVT---- 634

Imjin -----------T----EDQS-----IF-------PL-QST-VKSVFSYQLLLAV-TQKMKVCALFDNLRYLIPSCT---- 631

RVFV ----PSW----N----FVGNLFRSSDS----------AAMDASYMGKLSLLTLL-EDKATTEELQTIARYIIMEGF---- 679

Uukuniemi ----PFW----A----GNEKDFQT----------GK--Q-RANKMFKFCLLMLL-EDKARTEEIATLSRYVMMEGF---- 681

Peanut ------------------VDVFTCPEN-------KI--Q-YLQKILFSSVIIGT-VTKLSRMGIFDFMRYAGFLPL---- 918

Tomato ----------------EKVNVFSMTMT-------VK--Q-ILINIVFSSVMIGT-VTKLSRMGIFDFMRYAGFLPL---- 914

Lassa -----------S-------------EL-------EKELTH-IKLLTKMILVLILAHPSKRSQKFLQNLRYFIMAYV---- 653

Junin -----------H-------------EL----------MTM-TRPILRLLVLAVLCSPSKRNQTFLQGLRYFLMAYA---- 642

. : :

1. Multiple sequence alignment of spliceosomal Prp8 subunit

Legend

>S_cerevisiae >CAA80854.1

>S_pombe >NP_593861.1

>C_thermophilum >XP_006697396.1

>A_thaliana >NP_178124.2

>H_sapiens >NP_006436.3

>C_elegans >CCD66122.1

Structure-based alignment (extract)

S_cerevisiae EEYPVKVKVSYQKLLKNYVLNELHPTLPTNHNKTKLLKSLKNTKYFQQTTIDWVEAGLQL 571

S_pombe PNQPVKVRVSYQKLLKSHVMNKLHMAHPKSHTNRSLLRQLKNTKFFQSTSIDWVEAGLQV 519

C_thermophilum PKQPVKVRVSYQKLLKTYVLNELHRKRPKSMQKQSLLRTLKQTKFFQQTTIDWVEAGLQV 469

A_thaliana PAYPVKVRVSYQKLLKCYVLNELHHRPPKAQKKKHLFRSLAATKFFQSTELDWVEVGLQV 519

H_sapiens AGQPVKVRVSYQKLLKYYVLNALKHRPPKAQKKRYLFRSFKATKFFQSTKLDWVEVGLQV 496

C_elegans AGMPVKVRVSYQKLLKVFVLNALKHRPPKPQKRRYLFRSFKATKFFQTTTLDWVEAGLQV 488

****:******** .*:* *: *. . *:: : **:** * :****.***:

S_cerevisiae CRQGHNMLNLLIHRKGLTYLHLDYNFNLKPTKTLTTKERKKSRLGNSFHLMRELLKMMKL 631

S_pombe CRQGYNMLQLLIHRKGLTYLHLDYNCNLKPTKTLTTKERKKSRFGNAFHLMREILRLTKL 579

C_thermophilum CRQGFNMLNLLIHRKNLTYLHLDYNFNLKPVKTLTTKERKKSRFGNAFHLMREILRLTKL 529

A_thaliana CRQGYNMLNLLIHRKNLNYLHLDYNFNLKPVKTLTTKERKKSRFGNAFHLCREILRLTKL 579

H_sapiens CRQGYNMLNLLIHRKNLNYLHLDYNFNLKPVKTLTTKERKKSRFGNAFHLCREVLRLTKL 556

C_elegans LRQGYNMLNLLIHRKNLNYLHLDYNFNLKPVKTLTTKERKKSRFGNAFHLCREILRLTKL 548

***.***:******.*.******* ****.************:**:*** **:*:: **

S_cerevisiae IVDTHVQFRLGNVDAFQLADGIHYILNHIGQLTGIYRYKYKVMHQIRACKDLKHIIYYKF 691

S_pombe IVDSHVQYRLGNIDAYQLADGLHYIFNHVGQLTGMYRYKYRLMRQIRACKDFKHLIYYRF 639

C_thermophilum IVDAQVQYRLGNIDAFQLADGIHYAFNHVGQLTGMYRYKYKLMHQIRSCKDLKHLIYYRF 589

A_thaliana VVDANVQFRLGNVDAFQLADGLQYIFSHVGQLTGMYRYKYRLMRQIRMCKDLKHLIYYRF 639

H_sapiens VVDSHVQYRLGNVDAFQLADGLQYIFAHVGQLTGMYRYKYKLMRQIRMCKDLKHLIYYRF 616

C_elegans VVDAHVQYRLNNVDAYQLADGLQYIFAHVGQLTGMYRYKYKLMRQVRMCKDLKHLIYYRF 608

:**::**:**.*:**:*****::* : *:*****:*****::*:*:* ***:**:***:*
